# Overcoming barriers to primary care research in Japan: a call to action

**DOI:** 10.1016/j.lanwpc.2025.101523

**Published:** 2025-03-14

**Authors:** Thomas Rouyard, Emilie Yoda, Ryota Nakamura, Michiko Moriyama, Masako Ii, Maham Stanyon, Mei Endo, Koki Nakamura, Satoshi Kanke, Ryuki Kassai

**Affiliations:** a Hitotsubashi Institute for Advanced Study, Hitotsubashi University, Tokyo, Japan; b Graduate School of Public Health and Health Policy, City University of New York, New York, USA; c Department of Global Health and Development, London School of Hygiene and Tropical Medicine, London, UK; d Division of Nursing Science, Graduate School of Biomedical and Health Sciences, Hiroshima University, Hiroshima, Japan; e Graduate School of Economics, Hitotsubashi University, Tokyo, Japan; f Department of Community and Family Medicine, Fukushima Medical University, Fukushima, Japan; g World Organization of Family Doctors, Brussels, Belgium

In Japan, where primary care has historically been loosely defined and underutilized,^1^ efforts to strengthen its role within the healthcare system are gaining momentum.^2^^,^^3^ This shift is crucial as the country grapples with the pressures of a super-aged society,^4^ where a well-developed primary care system could help ease the growing financial strain on the healthcare system. While previous discussions have emphasized the need for policy reforms—particularly in medical education^5^—and the role of research in shaping these policies,^6^ far less attention has been given to the structural barriers that hinder primary care research. This commentary sheds light on these barriers by providing a first-hand account of major but largely undocumented obstacles that researchers face when conducting large-scale primary care studies in Japan. Drawing on our recent experience designing a randomized controlled trial (RCT), we offer empirical insights into real-world challenges that are often missing from theoretical discussions or expert recommendations. By highlighting these barriers and proposing actionable policy solutions, we aim to contribute to ongoing efforts to build a more evidence-informed and sustainable primary care system in Japan.

Japan’s medical landscape has long been dominated by organ specialists, with blurred distinctions between primary and secondary care.^1^ Family doctors—referred to here as general practitioners (GPs) for simplicity—lack a gatekeeping role, leading many patients to seek primary care from specialists at secondary care facilities. This lack of clear boundaries has hindered the development of primary care as an independent discipline. However, change is underway. The Japanese government has recognized the need to strengthen primary care to help control rising healthcare costs, valuing its patient-centered approach and integration of both clinical and social roles. Recent initiatives include promoting *kakaritsuke* physicians^3^—former organ specialists providing quasi-primary care in the community without formal GP training—and introducing fee incentives to discourage tertiary hospital use without referrals.^7^ Yet, these measures have not fostered the development of research-oriented primary care specialists meeting global standards. Consequently, studies remain largely hospital-based and organ-specific.^8^

The scarcity of primary care research in Japan is concerning as efforts to strengthen primary care progress. For instance, we identified only four RCTs conducted in primary care settings—where subject recruitment occurred in GP or *kakaritsuke* physician practices and/or the research involved GPs or *kakaritsuke* physicians—that were published in international peer-reviewed journals over the past decade (see search details in Appendix). In contrast, several high-income countries facing similar healthcare system sustainability challenges—largely publicly funded systems with ageing populations—have produced significantly more (Fig. 1). This broader lack of research, extending beyond RCTs,^9^ severely hampers the identification of (cost-)effective interventions and the development of evidence-based policies essential for building an equitable and sustainable primary care system.

**Fig. 1:**
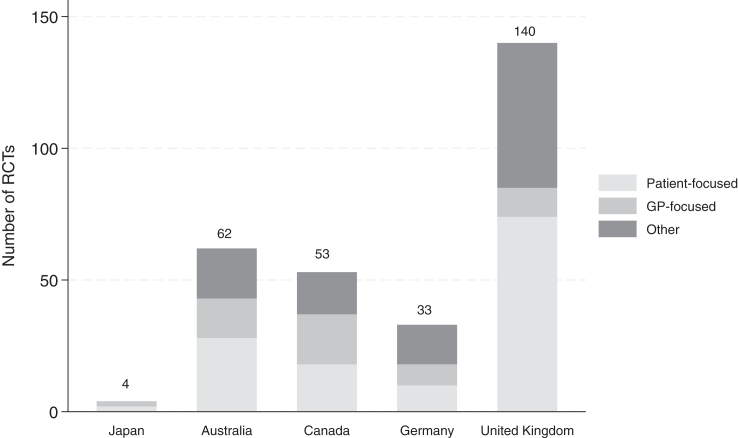
Number of randomized controlled trials conducted in primary care settings published in international peer-reviewed journals on or after 2015. *Note:***Patient-focused:** Interventions that are behavioural, educational, or informational, targeting patients. **GP-focused:** Interventions that are behavioural, educational, or informational, targeting general practitioners (GPs). **Other:** Studies evaluating new models of care, assessing the impact of new clinical guidelines, or contributing to research methodology. If multiple papers referred to the same trial (e.g., a study protocol and its corresponding results paper), only one was included. Papers published after 2015 were excluded if their primary findings had already been reported before 2015. **Primary care settings:** Studies were considered to be conducted in primary care if participant recruitment occurred in GP or *kakaritsuke* physician practices and/or if the research involved GPs or *kakaritsuke* physicians. Studies conducted in secondary care, specialized services (e.g., sexual health clinics, mental health services, physiotherapy, maternity care, nursing homes), pharmacies, or dental practices were excluded. However, exceptions were made for studies comparing models of care, such as those examining primary care versus secondary care. Studies conducted in multiple countries were excluded. To provide a standardized comparison, we calculated the number of outputs per 10 million inhabitants by dividing publication counts by each country’s population: Australia—23.2; United Kingdom—20.5; Canada—13.2; Germany—4.0; Japan—0.3.

Primary care research in Japan faces two major barriers. First, too few GPs have formal research training, and those who do often struggle with overwhelming workloads, leaving little time for research. These challenges were evident during our recent RCT, which aimed to evaluate a GP-led intervention in diabetes care.^10^ Despite two years of nationwide recruitment and extended deadlines to accommodate the unusual workloads caused by the COVID-19 crisis, we enrolled only 20 practices—well below the required sample size. While the pandemic initially hindered recruitment, challenges persisted even after routine care returned to normal capacity. Many GPs had limited research training and experience and feared the administrative burden, leading to initial hesitation about participation. Even when enthusiasm led to involvement, the study’s demands—such as long timelines and the effort required to integrate the intervention into routine consultations—frequently resulted in dropouts. Additionally, the limited pool of eligible GP practices, largely due to Japan’s underdeveloped GP system, further restricted recruitment. Despite minimal eligibility criteria—requiring only one GP certified by the Japan Primary Care Association (JPCA) per practice—few met this requirement. Of the 900 JPCA-certified GPs nationwide at the time of recruitment, only 167 had registered a clinic as their primary workplace, narrowing the pool of potential participants.

Second, Japan’s specialist-dominated healthcare system creates institutional and logistical barriers to primary care research. Leading funding bodies, such as the Japan Society for the Promotion of Science (JSPS), often exclude primary care from research funding categories. Ethics committees may also be skeptical of GP-led studies, particularly RCTs. For example, despite meeting international standards and passing peer review, our protocol faced resistance from two ethics committees before receiving approval from a third. In our case, methodological scrutiny—resulting in extensive paperwork for participating GP practices and pressures to modify our methods—took precedence over ethical considerations, prolonging the approval process and leading to the withdrawal of initially willing participants. Finally, infrastructural gaps, such as the absence of patient registration systems and standardized practices at the GP level, further complicate research efforts.

We believe progress can be made through targeted efforts in three key areas. First, cultivating a stronger research culture among GPs is essential. This can be achieved by offering accessible training in research design and methodology, encouraging GPs to develop practice-relevant research questions, and providing consistent support for their involvement in research activities. The establishment of the ‘general medicine’ specialty within Japan’s medical education system in 2018 presented an opportunity to foster this culture. Efforts have been made to integrate clinical research into GP training curricula, including mentorship programs and collaborations with academic institutions. For example, the JPCA, established in 2010 to support research and improve care quality,^2^ now offers courses aimed at continuing research capacity development. However, further steps are needed to refine the specialty’s definition and build broader recognition of primary care as an essential field.

While cultural recognition of primary care as a distinct specialty may take time, institutional changes—such as introducing dedicated funding schemes and strengthening Practice-Based Research Networks (PBRNs)—could accelerate progress by streamlining grant applications and research coordination.^11^ Additionally, fostering international collaborations, including bilateral funding partnerships with research agencies in countries with well-established primary care research cultures, and networks like the World Organization of Family Doctors (WONCA)^12^ and HTAsiaLink,^13^ would help build research capacity, secure funding, and promote knowledge exchange.^11^^,^^12^ Lastly, increasing the presence of primary care experts on research grant panels would ensure a more balanced evaluation process and expand funding opportunities for research directly addressing primary care needs.

Finally, addressing infrastructure challenges is critical. Strengthening Japanese PBRNs would not only ease recruitment challenges but also promote resource sharing and collective learning within the primary care community. In this regard, WONCA and other professional organizations have played a key role in developing PBRNs and supporting infrastructure improvements in other countries—such as the implementation of electronic health records for primary care in the Netherlands^11^—both of which could help address critical gaps in Japan. With the Ministry of Health moving toward centralizing patient records—currently limited to emergency use—there is an opportunity to expand this effort into a comprehensive patient registration system. Such a system would open new research opportunities, facilitate study implementation, and support the development of a sustainable and effective primary care research ecosystem in Japan.

## Contributors

TR and RK conceptualized the manuscript. EY conducted the literature search. TR drafted the manuscript. All authors substantially edited the manuscript. All authors have final responsibility for the decision to submit the manuscript for publication.

## Declaration of interests

All authors declare no competing interests.

## Appendix ASupplementary data

Appendix Tables A1–A6
