# Supplementary material for: Overcoming barriers to primary care research in Japan: a call to action
Source: Lancet Reg Health West Pac. 2025 Mar 14;56:101523. doi: 10.1016/j.lanwpc.2025.101523 (PMC11937693; doi:10.1016/j.lanwpc.2025.101523)
Supplement: Appendix Tables A1–A6 [file mmc1.docx]

**Supplementary Materials**

**Table of contents**

| **Table A1.** Search strategy (Pubmed) . | p.2 |
| --- | --- |
| **Table A2.** Reference list of randomized controlled trials conducted in primary care settings in Japan, 2015-2025 | p.3 |
| **Table A3.** Reference list of randomized controlled trials conducted in primary care settings in Australia, 2015-2025 | p.4 |
| **Table A4.** Reference list of randomized controlled trials conducted in primary care settings in Canada, 2015-2025 | p.9 |
| **Table A5.** Reference list of randomized controlled trials conducted in primary care settings in Germany, 2015-2025 | p.13 |
| **Table A6.** Reference list of randomized controlled trials conducted in primary care settings in the UK, 2015-2025 | p.16 |

**Table A1. Search strategy (Pubmed)**

| **Groups** | **Descriptors** | **Boolean operator** | **Field searched** |
| --- | --- | --- | --- |
| Theme | "primary care" OR "general practice" OR "general practitioner" OR "family medicine" OR "family doctor" | AND | [All] |
| Study design | RCT OR randomized OR randomised | AND | [Title/Abstract] |
| Country | Japan OR Australia OR Canada OR Germany OR (UK OR “United Kingdom”) | AND | [Title/Abstract] |

**Table A2. Reference list of randomized controlled trials conducted in primary care settings in Japan, 2015-2025**

| **Reference** | **Intervention focus*** |
| --- | --- |
| 1. Kanke, S., Kawai, T., Takasawa, N., Mashiyama, Y., Ishii, A., & Kassai, R. (2015). Interventions for body weight reduction in obese patients during short consultations: an open-label randomized controlled trial in the Japanese primary care setting. *Asia Pacific Family Medicine*, *14*, 1-12. | Patient-focused |
| 1. Noto, H., Tanizawa, Y., Aizawa, T., Sone, H., Yoshioka, N., Terauchi, Y., ... & Noda, M. (2016). Cluster‐randomized trial to improve the quality of diabetes management: the study for the efficacy assessment of the standard diabetes manual (SEAS‐DM). *Journal of diabetes investigation*, *7*(4), 539-543 | GP-focused |
| 1. Hayashino, Y., Suzuki, H., Yamazaki, K., Goto, A., Izumi, K., & Noda, M. (2016). A cluster randomized trial on the effect of a multifaceted intervention improved the technical quality of diabetes care by primary care physicians: The Japan Diabetes Outcome Intervention Trial‐2 (J‐DOIT 2). *Diabetic Medicine*, *33*(5), 599-608 | GP-focused |
| 1. Yamagata, K., Makino, H., Iseki, K., Ito, S., Kimura, K., Kusano, E., ... & Study Group for Frontier of Renal Outcome Modifications in Japan (FROM-J). (2016). Effect of behavior modification on outcome in early-to moderate-stage chronic kidney disease: a cluster-randomized trial. *PloS one*, *11*(3), e0151422 | Patient-focused |

***Patient-focused:** Interventions that are behavioral, educational, or informational, targeting patients. **GP-focused:** Interventions that are behavioral, educational, or informational, targeting general practitioners (GPs). **Other:** Studies evaluating new models of care, assessing the impact of new clinical guidelines, or contributing to research methodology.

**Note:** If multiple papers referred to the same trial (e.g., a study protocol and its corresponding results paper), only one was included. Papers published after 2015 were excluded if their primary findings had already been reported before 2015. **Primary care settings:** Studies were considered to be conducted in primary care if participant recruitment took place in GP or *kakaritsuke* physician practices and/or if the research involved GPs or *kakaritsuke* physicians. Studies conducted in secondary care, specialized services (e.g., sexual health clinics, mental health services, physiotherapy, maternity care, nursing homes), pharmacies, or dental practices were excluded. However, exceptions were made for studies comparing models of care, such as those examining primary care versus secondary care. Studies conducted in multiple countries were excluded.

**Table A3. Reference list of randomized controlled trials conducted in primary care settings in Australia, 2015-2025**

| **Reference** | **Intervention focus*** |
| --- | --- |
| 1. Fathima, P., Jones, M., D’Souza, R., Totterdell, J., Andric, N., Abbott, P., ... & Snelling, T. (2024). Financial incentives to motivate treatment for hepatitis C with direct acting antivirals among Australian adults (The Methodical evaluation and Optimisation of Targeted Incentives for Accessing Treatment of Early-stage hepatitis C: MOTIVATE-C): protocol for a dose-response randomised controlled study. *Trials*, *25*(1), 387. | Patient-focused |
| 1. Lawn, S., Shelby-James, T., Manger, S., Byrne, L., Fuss, B., Isaac, V., ... & Worley, P. (2024). Evaluation of lived experience Peer Support intervention for mental health service consumers in Primary Care (PS-PC): study protocol for a stepped-wedge cluster randomised controlled trial. *Trials*, *25*(1), 319. | Patient-focused |
| 1. McIntosh, J. G., Emery, J. D., Wood, A., Chondros, P., Goodwin, B. C., Trevena, J., ... & Jenkins, M. A. (2023). SMARTER screen protocol: a three-arm cluster randomised controlled trial of patient SMS messaging in general practice to increase participation in the Australian National Bowel Cancer Screening Program. *Trials*, *24*(1), 723. | Patient-focused |
| 1. Wallis, K. A., Donald, M., Horowitz, M., Moncrieff, J., Ware, R. S., Byrnes, J., ... & Scott, I. (2023). RELEASE (REdressing Long-tErm Antidepressant uSE): protocol for a 3-arm pragmatic cluster randomised controlled trial effectiveness-implementation hybrid type-1 in general practice. *Trials*, *24*(1), 615. | Patient-focused |
| 1. Currie, G. E., Totterdell, J., Bowland, G., Leeb, A., Peters, I., Blyth, C. C., ... & Snelling, T. L. (2023). The AuTOMATIC trial: a study protocol for a multi-arm Bayesian adaptive randomised controlled trial of text messaging to improve childhood immunisation coverage. *Trials*, *24*(1), 97. | Patient-focused |
| 1. Wood, A., Emery, J. D., Jenkins, M., Chondros, P., Campbell, T., Wenkart, E., ... & McIntosh, J. G. (2022). The SMART screen Trial: a randomised controlled trial investigating the efficacy of a GP-endorsed narrative SMS to increase participation in the Australian National Bowel Cancer Screening Program. *Trials*, *23*(1), 31. | Patient-focused |
| 1. Bonney, A., Metusela, C., Mullan, J., Barnett, S., Rhee, J., Kobel, C., & Batterham, M. (2021). Clinical and healthcare improvement through My Health Record usage and education in general practice (CHIME-GP): a study protocol for a cluster-randomised controlled trial. *Trials*, *22*(1), 569. | GP-focused |
| 1. Milton, S., McIntosh, J., Macrae, F., Chondros, P., Trevena, L., Jenkins, M., ... & Emery, J. (2021). An RCT of a decision aid to support informed choices about taking aspirin to prevent colorectal cancer and other chronic diseases: a study protocol for the SITA (Should I Take Aspirin?) trial. *Trials*, *22*, 1-17. | Patient-focused |
| 1. Walker, J. G., Macrae, F., Winship, I., Oberoi, J., Saya, S., Milton, S., ... & Emery, J. D. (2018). The use of a risk assessment and decision support tool (CRISP) compared with usual care in general practice to increase risk-stratified colorectal cancer screening: study protocol for a randomised controlled trial. *Trials*, *19*, 1-14. | Patient-focused |
| 1. Paul, C. L., Piterman, L., Shaw, J. E., Kirby, C., Forshaw, K. L., Robinson, J., ... & Sanson-Fisher, R. W. (2017). Poor uptake of an online intervention in a cluster randomised controlled trial of online diabetes education for rural general practitioners. *Trials*, *18*, 1-7. | GP-focused |
| 1. Emery, J. D., Murray, S. R., Walter, F. M., Martin, A., Goodall, S., Mazza, D., ... & Murchie, P. (2019). The Chest Australia Trial: a randomised controlled trial of an intervention to increase consultation rates in smokers at risk of lung cancer. *Thorax*, *74*(4), 362-370. | Patient-focused |
| 1. Peterson, G. M., Radford, J., Russell, G., Zwar, N., Mullan, J., Batterham, M., ... & Bonney, A. (2023). Cluster-randomised trial of the Effectiveness of Quality Incentive Payments in General Practice (EQuIP-GP): Prescribing of medicines outcomes. *Research in Social and Administrative Pharmacy*, *19*(5), 836-840. | GP-focused |
| 1. Trevena, L. J., Meiser, B., Mills, L., Dobbins, T., Mazza, D., Emery, J. D., ... & Naicker, S. (2022). Which Test Is Best? A Cluster-Randomized Controlled Trial of a Risk Calculator and Recommendations on Colorectal Cancer Screening Behaviour in General Practice. *Public Health Genomics*, *25*(5-6), 193-208. | Patient-focused |
| 1. Manski-Nankervis, J. A., Furler, J., O’Neal, D., Ginnivan, L., Thuraisingam, S., & Blackberry, I. (2017). Overcoming clinical inertia in insulin initiation in primary care for patients with type 2 diabetes: 24-month follow-up of the Stepping Up cluster randomised controlled trial. *Primary Care Diabetes*, *11*(5), 474-481. | Other |
| 1. Bajorek BV, Magin PJ, Hilmer SN, Krass I. Optimizing stroke prevention in patients with atrial fibrillation: a cluster-randomized controlled trial of a computerized antithrombotic risk assessment tool in Australian General Practice, 2012-2013. *Prev Chronic Dis* 2016;13:1–13. | GP-focused |
| 1. Fletcher S, Chondros P, Densley K, Murray E, Dowrick C, Coe A, et al. Matching depression management to severity prognosis in primary care: results of the Target-D randomised controlled trial. *Br J Gen Pract.* 2021; 71(703):e85–e94. | Patient-focused |
| 1. Sanci, L., Chondros, P., Sawyer, S., Pirkis, J., Ozer, E., Hegarty, K., ... & Patton, G. (2015). Responding to young people’s health risks in primary care: a cluster randomised trial of training clinicians in screening and motivational interviewing. *PloS one*, *10*(9), e0137581. | GP-focused |
| 1. Abramson, M. J., Schattner, R. L., Holton, C., Simpson, P., Briggs, N., Beilby, J., ... & Massie, R. J. (2015). Spirometry and regular follow‐up do not improve quality of life in children or adolescents with asthma: cluster randomized controlled trials. *Pediatric pulmonology*, *50*(10), 947-954. | Other |
| 1. Rebbeck, T., Bandong, A. N., Leaver, A., Ritchie, C., Armfield, N., Arora, M., ... & Sterling, M. (2023). Implementation of a risk-stratified, guideline-based clinical pathway of care to improve health outcomes following whiplash injury (Whiplash ImPaCT): a multicentre, randomized, controlled trial. *Pain*, *164*(10), 2216-2227. | Other |
| 1. Fletcher, S., Spittal, M. J., Chondros, P., Palmer, V. J., Chatterton, M. L., Densley, K., ... & Gunn, J. (2021). Clinical efficacy of a decision support tool (Link-me) to guide intensity of mental health care in primary practice: a pragmatic stratified randomised controlled trial. *The Lancet Psychiatry*, *8*(3), 202-214. | Patient-focused |
| 1. Guy, R. J., Ward, J., Causer, L. M., Natoli, L., Badman, S. G., Tangey, A., ... & Kaldor, J. M. (2018). Molecular point-of-care testing for chlamydia and gonorrhoea in Indigenous Australians attending remote primary health services (TTANGO): a cluster-randomised, controlled, crossover trial. *The Lancet Infectious Diseases*, *18*(10), 1117-1126. | Other |
| 1. Furler, J., O'Neal, D., Speight, J., Blackberry, I., Manski-Nankervis, J. A., Thuraisingam, S., ... & Best, J. (2020). Use of professional-mode flash glucose monitoring, at 3-month intervals, in adults with type 2 diabetes in general practice (GP-OSMOTIC): a pragmatic, open-label, 12-month, randomised controlled trial. *The Lancet Diabetes & Endocrinology*, *8*(1), 17-26. | Other |
| 1. Hocking, J. S., Temple-Smith, M., Guy, R., Donovan, B., Braat, S., Law, M., ... & Low, N. (2018). Population effectiveness of opportunistic chlamydia testing in primary care in Australia: a cluster-randomised controlled trial. *The Lancet*, *392*(10156), 1413-1422. | Other |
| 1. Dodd, N., Carey, M. L., Mansfield, E., & Oldmeadow, C. (2017). Testing the effectiveness of a primary care intervention to improve uptake of colorectal cancer screening: A randomized controlled trial protocol. *JMIR Research Protocols*, *6*(5), e7432. | Patient-focused |
| 1. Chidwick, K., Myton, R., Rodgers, A., Jun, M., Dartnell, J., Balcomb, A., & Dore, G. (2022). A cluster randomized controlled trial of a MedicineInsight Educational Quality Improvement Programme to improve the diagnosis and treatment of chronic hepatitis C in general practice (the EQUIP‐HEPC trial). *Journal of Viral Hepatitis*, *29*(2), 135-146. | GP-focused |
| 1. Kinchin, I., Kelley, S., Meshcheriakova, E., Viney, R., Mann, J., Thompson, F., & Strivens, E. (2022). Cost-effectiveness of a community-based integrated care model compared with usual care for older adults with complex needs: a stepped-wedge cluster-randomised trial. *Integrated healthcare journal*, *4*(1). | Other |
| 1. Mazza, D., Chakraborty, S., Camões-Costa, V., Kenardy, J., Brijnath, B., Mortimer, D., ... & Collie, A. (2021). Implementing work-related Mental health guidelines in general PRacticE (IMPRovE): a protocol for a hybrid III parallel cluster randomised controlled trial. *Implementation Science*, *16*, 1-11. | GP-focused |
| 1. Clemson, L., Mackenzie, L., Roberts, C., Poulos, R., Tan, A., Lovarini, M., ... & White, F. (2017). Integrated solutions for sustainable fall prevention in primary care, the iSOLVE project: a type 2 hybrid effectiveness-implementation design. *Implementation science*, *12*, 1-12. | GP-focused |
| 1. Russell, A. W., Donald, M., Borg, S. J., Zhang, J., Burridge, L. H., Ware, R. S., ... & Jackson, C. L. (2019). Clinical outcomes of an integrated primary–secondary model of care for individuals with complex type 2 diabetes: a non-inferiority randomised controlled trial. *Diabetologia*, 62, 41-52. | Other |
| 1. Barbaro, J., Masi, A., Gilbert, M., Nair, R., Abdullahi, I., Descallar, J., ... & Eapen, V. (2021). A multistate trial of an early surveillance program for autism within general practices in Australia. *Frontiers in Pediatrics*, *9*, 640359. | Other |
| 1. Vuong, K., Armstrong, B. K., McGeechan, K., & Cust, A. E. (2019). Personalized melanoma risk assessments and tailored prevention advice: a pragmatic randomized controlled trial in Australian general practice. *Family Practice*, *36*(2), 237-246. | Patient-focused |
| 1. Zwar, N. A., Richmond, R. L., Halcomb, E. J., Furler, J. S., Smith, J. P., Hermiz, O., ... & Borland, R. (2015). Quit in general practice: a cluster randomized trial of enhanced in-practice support for smoking cessation. *Family Practice*, 32(2), 173-180. | Patient-focused |
| 1. Charles, D., Heal, C. F., Delpachitra, M., Wohlfahrt, M., Kimber, D., Sullivan, J., ... & Buttner, P. (2017). Alcoholic versus aqueous chlorhexidine for skin antisepsis: the AVALANCHE trial. *Cmaj*, *189*(31), E1008-E1016. | Other |
| 1. Bonney, A., Kobel, C., Mullan, J., Metusela, C., Rhee, J. J., Barnett, S., & Batterham, M. (2023). Randomised trial of general practitioner online education for prescribing and test ordering. *BMJ Open Quality*, *12*(4), e002351. | GP-focused |
| 1. Singleton, A. C., Partridge, S. R., Hyun, K. K., Mitchell, C., Raeside, R., Hafiz, N., ... & Redfern, J. (2024). Text message intervention delivered from Australian general practices to improve breast cancer survivors’ physical activity and cardiovascular risk factors: protocol for the EMPOWER-SMS-GP effectiveness implementation randomised controlled trial. *BMJ open*, *14*(12), e090984. | Patient-focused |
| 1. Coe, A., Gunn, J., Allnutt, Z., & Kaylor-Hughes, C. (2024). Understanding Australian general practice patients’ decisions to deprescribe antidepressants in the WiserAD trial: a realist informed approach. *BMJ open*, *14*(2), e078179. | Patient-focused |
| 1. Cross, A. J., Geethadevi, G. M., Magin, P., Baker, A. L., Bonevski, B., Godbee, K., ... & George, J. (2023). A novel, multidomain, primary care nurse-led and mHealth-assisted intervention for dementia risk reduction in middle-aged adults (HAPPI MIND): study protocol for a cluster randomised controlled trial. *BMJ open*, *13*(12), e073709. | Patient-focused |
| 1. Hocking, J. S., Watson, C., Chondros, P., Sawyer, S. M., Ride, J., Temple-Smith, M., ... & Sanci, L. (2023). Will a fee-for-service payment for a young people’s health assessment in general practice increase the detection of health risk behaviours and health conditions? Protocol for a cluster randomised controlled trial (RAd Health Trial). *BMJ open*, *13*(8), e074154. | GP-focused |
| 1. Mazza, D., Shankar, M., Botfield, J. R., Moulton, J. E., Chakraborty, S. P., Black, K., ... & Norman, W. V. (2023). Improving rural and regional access to long-acting reversible contraception and medical abortion through nurse-led models of care, task-sharing and telehealth (ORIENT): a protocol for a stepped-wedge pragmatic cluster-randomised controlled trial in Australian general practice. *BMJ open*, *13*(3), e065137. | Other |
| 1. Khano, S., Sanci, L., Woolfenden, S., Zurynski, Y., Dalziel, K., Liaw, S. T., ... & Hiscock, H. (2022). Strengthening Care for Children (SC4C): protocol for a stepped wedge cluster randomised controlled trial of an integrated general practitioner-paediatrician model of primary care. *BMJ open*, *12*(9), e063449. | Other |
| 1. Marukutira, T., Moore, K. P., Hellard, M., Richmond, J., Turner, K., Pedrana, A. E., ... & Doyle, J. (2022). Randomised controlled trial of active case management to link hepatitis C notifications to treatment in Tasmania, Australia: a study protocol. *BMJ open*, *12*(3), e056120. | Other |
| 1. Taft, A., Young, F., Hegarty, K., Yelland, J., Mazza, D., Boyle, D., ... & Feder, G. (2021). HARMONY: a pragmatic cluster randomised controlled trial of a culturally competent systems intervention to prevent and reduce domestic violence among migrant and refugee families in general practice: study protocol. *BMJ open*, *11*(7), e046431. | Patient-focused |
| 1. Ellerton, K., Tharmarajah, H., Medres, R., Brown, L., Ringelblum, D., Vogel, K., ... & Craig, S. (2020). The VRIMM study: Virtual Reality for IMMunisation pain in young children—protocol for a randomised controlled trial. *BMJ open*, *10*(8), e038354. | Patient-focused |
| 1. Carey, M., Sanson-Fisher, R., Zwar, N., Mazza, D., Meadows, G., Piterman, L., ... & Kelly, B. (2020). Improving depression outcomes among Australian primary care patients: protocol for a cluster randomised controlled trial. *BMJ open*, *10*(2), e032057. | GP-focused |
| 1. Pond, D., Mate, K., Stocks, N., Gunn, J., Disler, P., Magin, P., ... & Brodaty, H. (2018). Effectiveness of a peer-mediated educational intervention in improving general practitioner diagnostic assessment and management of dementia: a cluster randomised controlled trial. *BMJ open*, *8*(8), e021125. | GP-focused |
| 1. Parker, S. M., Stocks, N., Nutbeam, D., Thomas, L., Denney-Wilson, E., Zwar, N., ... & Harris, M. F. (2018). Preventing chronic disease in patients with low health literacy using eHealth and teamwork in primary healthcare: protocol for a cluster randomised controlled trial. *BMJ open*, *8*(6), e023239. | Patient-focused |
| 1. Kim, S., McMaster, M., Torres, S., Cox, K. L., Lautenschlager, N., Rebok, G. W., ... & Anstey, K. J. (2018). Protocol for a pragmatic randomised controlled trial of Body Brain Life—General Practice and a Lifestyle Modification Programme to decrease dementia risk exposure in a primary care setting. *BMJ open*, *8*(3), e019329. | Patient-focused |
| 1. Liang, J., Abramson, M. J., Zwar, N., Russell, G., Holland, A. E., Bonevski, B., ... & George, J. (2017). Interdisciplinary model of care (RADICALS) for early detection and management of chronic obstructive pulmonary disease (COPD) in Australian primary care: study protocol for a cluster randomised controlled trial. *BMJ open*, *7*(9), e016985. | Other |
| 1. Mazza, D., Black, K., Taft, A., Lucke, J., McGeechan, K., Haas, M., ... & Peipert, J. F. (2016). Increasing the uptake of long-acting reversible contraception in general practice: the Australian Contraceptive ChOice pRoject (ACCORd) cluster randomised controlled trial protocol. *BMJ open*, *6*(10), e012491. | GP-focused |
| 1. Paul, C., Rose, S., Hensley, M., Pretto, J., Hardy, M., Henskens, F., ... & Carey, M. (2016). Examining uptake of online education on obstructive sleep apnoea in general practitioners: a randomised trial. *BMC Research Notes*, *9*, 1-6. | GP-focused |
| 1. Faruqi, N., Stocks, N., Spooner, C., El Haddad, N., & Harris, M. F. (2015). Research protocol: management of obesity in patients with low health literacy in primary health care. *BMC obesity*, *2*, 1-8. | Other |
| 1. Reed, R. L., Roeger, L., & Kaambwa, B. (2024). Two-year follow-up of a clustered randomised controlled trial of a multicomponent general practice intervention for people at risk of poor health outcomes. *BMC Health Services Research*, *24*(1), 488. | Other |
| 1. Avent, M. L., Hansen, M. P., Gilks, C., Del Mar, C., Halton, K., Sidjabat, H., ... & Van Driel, M. L. (2016). General Practitioner Antimicrobial Stewardship Programme Study (GAPS): protocol for a cluster randomised controlled trial. *BMC family practice*, *17*, 1-9. | GP-focused |
| 1. Schumacher, T. L., Herbert, J., May, J., Ramanathan, S., Brown, L. J., Guppy, M., ... & Collins, C. E. (2023). HealthyRHearts-reducing cholesterol in rural adults via telehealth-based medical nutrition therapy: protocol for a cluster randomised controlled trial. *BMC Cardiovascular Disorders*, *23*(1), 297. | Patient-focused |
| 1. Guppy, M., Glasziou, P., Jones, M., Beller, E., Shaw, J. E., Barr, E., & Doust, J. (2024). Kidney trajectory charts improve GP management of patients with reduced kidney function: a randomised controlled vignette study. *BJGP open*. | GP-focused |
| 1. Dodd, N., Carey, M., Mansfield, E., Oldmeadow, C., & Evans, T. J. (2019). Testing the effectiveness of a general practice intervention to improve uptake of colorectal cancer screening: a randomised controlled trial. *Australian and New Zealand journal of public health*, 43(5), 464-469. | Patient-focused |
| 1. Ewald, B., Stacey, F., Johnson, N., Plotnikoff, R. C., Holliday, E., Brown, W., & James, E. L. (2018). Physical activity coaching by Australian Exercise Physiologists is cost effective for patients referred from general practice. *Australian and New Zealand Journal of Public Health*, 42(1), 12-15. | Patient-focused |
| 1. MacDonald, E. J., Geller, S., Sibanda, N., Stevenson, K., Denmead, L., Adcock, A., ... & Lawton, B. (2021). Reaching under‐screened/never‐screened indigenous peoples with human papilloma virus self‐testing: a community‐based cluster randomised controlled trial. *Australian and New Zealand Journal of Obstetrics and Gynaecology,* 61(1), 135-141. | Patient-focused |
| 1. Hunter, D. J., Bowden, J. L., Hinman, R. S., Egerton, T., Briggs, A. M., Bunker, S. J., ... & PARTNER Study Team. (2023). Effectiveness of a new service delivery model for management of knee osteoarthritis in primary care: a cluster randomized controlled trial. *Arthritis Care & Research*, *75*(6), 1320-1332. | Other |
| 1. James, E. L., Ewald, B. D., Johnson, N. A., Stacey, F. G., Brown, W. J., Holliday, E. G., ... & Plotnikoff, R. C. (2017). Referral for expert physical activity counseling: a pragmatic RCT. *American journal of preventive medicine*, *53*(4), 490-499. | Patient-focused |
| 1. Goode, A. D., Winkler, E. A., Reeves, M. M., & Eakin, E. G. (2015). Relationship between intervention dose and outcomes in living well with diabetes—a randomized trial of a telephone-delivered lifestyle-based weight loss intervention. *American Journal of Health Promotion*, *30*(2), 120-129. | Patient-focused |
| 1. Gnanenthiran, S. R., Tan, I., Atkins, E. R., Avolio, A., Bennett, B., Chapman, N., ... & Schutte, A. E. (2023). Transforming blood pressure control in primary care through a novel remote decision support strategy based on wearable blood pressure monitoring: The NEXTGEN-BP randomized trial protocol. *American Heart Journal*, *265*, 50-58. | Other |
| 1. Attia, J. R., Holliday, E., Weaver, N., Peel, R., Fleming, K. C., Hure, A., ... & Acharya, S. (2022). The effect of zinc supplementation on glucose homeostasis: a randomised double-blind placebo-controlled trial. *Acta Diabetologica*, *59*(7), 965-975. | Other |

***Patient-focused:** Interventions that are behavioral, educational, or informational, targeting patients. **GP-focused:** Interventions that are behavioral, educational, or informational, targeting general practitioners (GPs). **Other:** Studies evaluating new models of care, assessing the impact of new clinical guidelines, or contributing to research methodology.

**Note:** If multiple papers referred to the same trial (e.g., a study protocol and its corresponding results paper), only one was included. Papers published after 2015 were excluded if their primary findings had already been reported before 2015. **Primary care settings:** Studies were considered to be conducted in primary care if participant recruitment took place in GP practices and/or if the research involved GPs. Studies conducted in secondary care, specialized services (e.g., sexual health clinics, mental health services, physiotherapy, maternity care, nursing homes), pharmacies, or dental practices were excluded. However, exceptions were made for studies comparing models of care, such as those examining primary care versus secondary care. Studies conducted in multiple countries were excluded.

**Table A4. Reference list of randomized controlled trials conducted in primary care settings in Canada, 2015-2025**

| **Reference** | **Intervention focus*** |
| --- | --- |
| 1. Rae, S., Maguire, J., Aglipay, M., Barwick, M., Danavan, K., Haines, J., ... & Birken, C. (2023). Randomized controlled trial evaluating a virtual parenting intervention for young children at risk of obesity: study protocol for Parenting Addressing Early Years Intervention with Coaching Visits in Toronto (PARENT) trial. *Trials*, *24*(1), 8. | Patient-focused |
| 1. Cox, J., Hamilton, L., Thabane, L., Foster, G., MacKillop, J., Xie, F., ... & IMPACT-AF Investigators. (2024). Computerized clinical decision support to improve stroke prevention therapy in primary care management of atrial fibrillation: a cluster randomized trial. *American Heart Journal*, 273, 102-110. | GP-focused |
| 1. Mangin, D., Lamarche, L., Oliver, D., Bomze, S., Borhan, S., Browne, T., ... & Price, D. (2020). Health TAPESTRY Ontario: protocol for a randomized controlled trial to test reproducibility and implementation. *Trials*, *21*, 1-14. | Other |
| 1. Turner, J. P., Caetano, P., & Tannenbaum, C. (2019). Leveraging policy to reduce chronic opioid use by educating and empowering community dwelling adults: a study protocol for the TAPERING randomized controlled trial. *Trials*, *20*, 1-9. | Patient-focused |
| 1. Li, A. H., Garg, A. X., Prakash, V., Grimshaw, J. M., Taljaard, M., Mitchell, J., ... & Presseau, J. (2017). Promoting deceased organ and tissue donation registration in family physician waiting rooms (RegisterNow-1 trial): study protocol for a pragmatic, stepped-wedge, cluster randomized controlled registry. *Trials*, *18*, 1-13. | Patient-focused |
| 1. You, J. J., Liu, Y., Kirby, J., Vora, P., & Moayyedi, P. (2015). Virtual colonoscopy, optical colonoscopy, or fecal occult blood testing for colorectal cancer screening: results of a pilot randomized controlled trial. *Trials*, *16*, 1-6. | Other |
| 1. Persaud, N., Bedard, M., Boozary, A., Glazier, R. H., Gomes, T., Hwang, S. W., ... & Carefully seLected and Easily Accessible at No Charge Medications (CLEAN Meds) study team. (2021). Adherence at 2 years with distribution of essential medicines at no charge: The CLEAN Meds randomized clinical trial. *PLoS Medicine*, *18*(5), e1003590. | Patient-focused |
| 1. Minian, N., Lingam, M., Moineddin, R., Thorpe, K. E., Veldhuizen, S., Dragonetti, R., ... & Selby, P. (2022). The impact of a clinical decision support system for addressing physical activity and healthy eating during Smoking Cessation Treatment: hybrid type I randomized controlled trial. *Journal of Medical Internet Research*, *24*(9), e37900. | Patient-focused |
| 1. Minian, N., Ahad, S., Ivanova, A., Veldhuizen, S., Zawertailo, L., Ravindran, A., ... & Selby, P. (2021). The effectiveness of generic emails versus a remote knowledge broker to integrate mood management into a smoking cessation programme in team-based primary care: a cluster randomised trial. *Implementation science*, *16*, 1-15. | GP-focused |
| 1. Wallack, E. M., Harris, C., Ploughman, M., & Butler, R. (2018). Telegerontology as a novel approach to address health and safety by supporting community-based rural dementia care triads: randomized controlled trial protocol. *JMIR research protocols*, *7*(2), e8744. | Other |
| 1. Vaisson, G., Witteman, H. O., Bouck, Z., Bravo, C. A., Desveaux, L., Llovet, D., ... & Ivers, N. M. (2018). Testing behavior change techniques to encourage primary care physicians to access cancer screening audit and feedback reports: protocol for a factorial randomized experiment of email content. *JMIR research protocols*, *7*(2), e9090. | GP-focused |
| 1. Avis, J. L., Cave, A. L., Donaldson, S., Ellendt, C., Holt, N. L., Jelinski, S., ... & Ball, G. D. (2015). Working with parents to prevent childhood obesity: protocol for a primary care-based eHealth study. *JMIR research protocols*, *4*(1), e4147. | Patient-focused |
| 1. Gray, C. S., Gravesande, J., Hans, P. K., Nie, J. X., Sharpe, S., Loganathan, M., ... & Cott, C. (2019). Using exploratory trials to identify relevant contexts and mechanisms in complex electronic health interventions: evaluating the electronic patient-reported outcome tool. *JMIR Formative Research*, *3*(1), e11950. | Other |
| 1. Lear, S. A., Norena, M., Banner, D., Whitehurst, D. G., Gill, S., Burns, J., ... & Singer, J. (2021). Assessment of an Interactive Digital Health–Based Self-management Program to Reduce Hospitalizations Among Patients With Multiple Chronic Diseases: A Randomized Clinical Trial. *JAMA Network Open*, *4*(12), e2140591-e2140591. | Patient-focused |
| 1. Schwartz, K. L., Ivers, N., Langford, B. J., Taljaard, M., Neish, D., Brown, K. A., ... & Garber, G. (2021). Effect of antibiotic-prescribing feedback to high-volume primary care physicians on number of antibiotic prescriptions: a randomized clinical trial. *JAMA Internal Medicine*, *181*(9), 1165-1173. | GP-focused |
| 1. Aglipay, M., Birken, C. S., Parkin, P. C., Loeb, M. B., Thorpe, K., Chen, Y., ... & TARGet Kids! Collaboration. (2017). Effect of high-dose vs standard-dose wintertime vitamin D supplementation on viral upper respiratory tract infections in young healthy children. *Jama*, *318*(3), 245-254. | Other |
| 1. Parkin, P. C., Borkhoff, C. M., Macarthur, C., Abdullah, K., Birken, C. S., Fehlings, D., ... & Zajdman, M. (2021). Randomized trial of oral iron and diet advice versus diet advice alone in young children with nonanemic iron deficiency. *The Journal of Pediatrics*, *233*, 233-240. | Other |
| 1. Mah, S. J., Brotto, L. A., Bryce, M., Keast, S., Albert, A., & Lee, M. (2024). A Parallel-Group, Randomized Trial Examining Impact of Colposcopy Results Delivery by a Nurse Liaison on Patient-Reported Outcomes and Adherence. *Journal of Obstetrics and Gynaecology Canada*, *46*(12), 102668. | Other |
| 1. Stamenova, V., Nguyen, M., Onabajo, N., Merritt, R., Sutakovic, O., Mossman, K., ... & Bhattacharyya, O. (2023). Mailed letter versus phone call to increase diabetic-related retinopathy screening engagement by patients in a team-based primary care practice: prospective, single-masked, randomized trial. *Journal of Medical Internet Research*, *25*, e37867. | Patient-focused |
| 1. Wayne, N., Perez, D. F., Kaplan, D. M., & Ritvo, P. (2015). Health coaching reduces HbA1c in type 2 diabetic patients from a lower-socioeconomic status community: a randomized controlled trial. *Journal of medical Internet research*, *17*(10), e224. | Patient-focused |
| 1. Bareil, C., Duhamel, F., Lalonde, L., Goudreau, J., Hudon, E., Lussier, M. T., ... & Lalonde, G. (2015). Facilitating implementation of interprofessional collaborative practices into primary care: A trilogy of driving forces. *Journal of Healthcare Management*, *60*(4), 287-300. | Other |
| 1. Boekhout, A. H., Maunsell, E., Pond, G. R., Julian, J. A., Coyle, D., Levine, M. N., ... & FUPII Trial Investigators. (2015). A survivorship care plan for breast cancer survivors: extended results of a randomized clinical trial. *Journal of Cancer Survivorship*, *9*, 683-691. | Other |
| 1. Espinet, S. D., Gotovac, S., Knight, S., Zwarenstein, M., Lingard, L., & Steele, M. (2018). A study protocol for the “practitioner training in child and adolescent psychiatry” cluster-randomized pilot study. *Journal of the Canadian Academy of Child and Adolescent Psychiatry*, *27*(4), 236. | Other |
| 1. Tamblyn, R., Ernst, P., Winslade, N., Huang, A., Grad, R., Platt, R. W., ... & Eguale, T. (2015). Evaluating the impact of an integrated computer-based decision support with person-centered analytics for the management of asthma in primary care: a randomized controlled trial. *Journal of the American Medical Informatics Association*, *22*(4), 773-783. | GP-focused |
| 1. Kiran, T., Davie, S., Moineddin, R., & Lofters, A. (2018). Mailed letter versus phone call to increase uptake of cancer screening: a pragmatic, randomized trial. *The Journal of the American Board of Family Medicine*, *31*(6), 857-868. | Patient-focused |
| 1. Minian, N., Baliunas, D., Noormohamed, A., Zawertailo, L., Giesbrecht, N., Hendershot, C. S., ... & Selby, P. L. (2019). The effect of a clinical decision support system on prompting an intervention for risky alcohol use in a primary care smoking cessation program: a cluster randomized trial. *Implementation science*, *14*, 1-10. | GP-focused |
| 1. Greiver, M., Dahrouge, S., O’Brien, P., Manca, D., Lussier, M. T., Wang, J., ... & Farrell, B. (2019). Improving care for elderly patients living with polypharmacy: protocol for a pragmatic cluster randomized trial in community-based primary care practices in Canada. *Implementation Science*, *14*, 1-15. | GP-focused |
| 1. Tamblyn, R., Winslade, N., Qian, C. J., Moraga, T., & Huang, A. (2018). What is in your wallet? A cluster randomized trial of the effects of showing comparative patient out-of-pocket costs on primary care prescribing for uncomplicated hypertension. *Implementation Science*, *13*, 1-14. | GP-focused |
| 1. Minian, N., Baliunas, D., Zawertailo, L., Noormohamed, A., Giesbrecht, N., Hendershot, C. S., ... & Selby, P. L. (2017). Combining alcohol interventions with tobacco addictions treatment in primary care—the COMBAT study: a pragmatic cluster randomized trial. *Implementation Science*, *12*, 1-8. | GP-focused |
| 1. Dolovich, L., Oliver, D., Lamarche, L., Thabane, L., Valaitis, R., Agarwal, G., ... & Price, D. (2019). Combining volunteers and primary care teamwork to support health goals and needs of older adults: a pragmatic randomized controlled trial. *Cmaj*, *191*(18), E491-E500. | Other |
| 1. Liddy, C., Hogg, W., Singh, J., Taljaard, M., Russell, G., Deri Armstrong, C., ... & Grimshaw, J. M. (2015). A real-world stepped wedge cluster randomized trial of practice facilitation to improve cardiovascular care. *Implementation Science*, *10*, 1-11. | GP-focused |
| 1. Godwin, M., Gadag, V., Pike, A., Pitcher, H., Parsons, K., McCrate, F., ... & Miller, R. (2016). A randomized controlled trial of the effect of an intensive 1-year care management program on measures of health status in independent, community-living old elderly: the Eldercare project. *Family practice*, *33*(1), 37-41. | Other |
| 1. Klimas, J., Hamilton, M. A., Carney, G., Cooper, I. R., Croteau, N. S., Dong, H., ... & McCracken, R. (2021). Individualized prescribing portraits to reduce inappropriate initiation of opioid analgesics to opioid naïve patients in primary care: Protocol for a randomized controlled trial. *Contemporary Clinical Trials*, *107*, 106462. | GP-focused |
| 1. Fortin, M., Chouinard, M. C., Dubois, M. F., Bélanger, M., Almirall, J., Bouhali, T., & Sasseville, M. (2016). Integration of chronic disease prevention and management services into primary care: a pragmatic randomized controlled trial (PR1MaC). *Canadian Medical Association Open Access Journal*, *4*(4), E588-E598. | Patient-focused |
| 1. Okpechi, I. G., Zaidi, D., Ye, F., Fradette, M., Schick-Makaroff, K., Berendonk, C., ... & Bello, A. K. (2022). Telemonitoring and case management for hypertensive and remote-dwelling patients with chronic kidney disease—the Telemonitoring for Improved Kidney Outcomes Study (TIKO): a clinical research protocol. *Canadian Journal of Kidney Health and Disease*, *9*, 20543581221077500. | Other |
| 1. Nash, D. M., Ivers, N. M., Young, J., Jaakkimainen, R. L., Garg, A. X., & Tu, K. (2017). Improving care for patients with or at risk for chronic kidney disease using electronic medical record interventions: A pragmatic cluster-randomized trial protocol. *Canadian Journal of Kidney Health and Disease*, *4*, 2054358117699833. | GP-focused |
| 1. Dawes, D., Ashe, M., Campbell, K., Cave, D., Elley, C. R., Kaczorowski, J., ... & Dawes, M. (2015). Preventing diabetes in primary care: a feasibility cluster randomized trial. *Canadian journal of diabetes*, *39*(2), 111-116. | Patient-focused |
| 1. Howard, M., Elston, D., Borhan, S., Hafid, A., Arora, N., Forbes, R., ... & Heyland, D. K. (2022). Randomised trial of a serious illness decision aid (Plan Well Guide) for patients and their substitute decision-makers to improve engagement in advance care planning. *BMJ Supportive & Palliative Care*, *12*(1), 99-106. | Patient-focused |
| 1. Carney, G., Maclure, M., Patrick, D. M., Otte, J., Ambasta, A., Thompson, W., & Dormuth, C. (2024). Pragmatic randomised trial assessing the impact of peer comparison and therapeutic recommendations, including repetition, on antibiotic prescribing patterns of family physicians across British Columbia for uncomplicated lower urinary tract infections. *BMJ Quality & Safety*. | GP-focused |
| 1. Silverberg, N. D., Otamendi, T., Brasher, P. M., Brubacher, J. R., Li, L. C., Lizotte, P. P., ... & Archambault, P. (2022). Effectiveness of a guideline implementation tool for supporting management of mental health complications after mild traumatic brain injury in primary care: protocol for a randomised controlled trial. *BMJ open*, *12*(6), e062527. | GP-focused |
| 1. Hussey, A. J., McKelvie, R. S., Ferrone, M., To, T., Fisk, M., Singh, D., ... & Licskai, C. (2022). Primary care-based integrated disease management for heart failure: a study protocol for a cluster randomised controlled trial. *BMJ open*, *12*(5), e058608. | Patient-focused |
| 1. Alvarez, E., Qutob, M., Mbuagbaw, L., Lavis, J., Lokker, C., Walli-Attaei, M., ... & Fortuna, J. (2019). Feasibility and implementation of a healthy lifestyles program in a community setting in Ontario, Canada: protocol for a pragmatic mixed methods pilot study. *BMJ open*, *9*(10), e031298. | Patient-focused |
| 1. Persaud, N., Laupacis, A., Azarpazhooh, A., Birken, C., Hoch, J. S., Isaranuwatchai, W., ... & Parkin, P. (2018). Xylitol for the prevention of acute otitis media episodes in children aged 2–4 years: protocol for a pragmatic randomised controlled trial. *BMJ open*, *8*(8), e020941. | Other |
| 1. Schwartz, K. L., Shuldiner, J., Langford, B. J., Brown, K. A., Schultz, S. E., Leung, V., ... & Ivers, N. (2024). Mailed feedback to primary care physicians on antibiotic prescribing for patients aged 65 years and older: pragmatic, factorial randomised controlled trial. *bmj*, *385*. | GP-focused |
| 1. Roberge, P., Provencher, M. D., Gosselin, P., Vasiliadis, H. M., Gaboury, I., Benoit, A., ... & Norton, P. J. (2018). A pragmatic randomized controlled trial of group transdiagnostic cognitive-behaviour therapy for anxiety disorders in primary care: study protocol. *BMC psychiatry*, *18*, 1-14. | Patient-focused |
| 1. Manca, D. P., Fernandes, C., Lofters, A., Aubrey-Bassler, K., Shea-Budgell, M., Campbell-Scherer, D., ... & Grunfeld, E. (2023). Results from the BETTER WISE trial: a pragmatic cluster two arm parallel randomized controlled trial for primary prevention and screening in primary care during the COVID-19 pandemic. *BMC Primary Care*, *24*(1), 200. | Patient-focused |
| 1. Singer, A., Kosowan, L., Abrams, E. M., Katz, A., Lix, L., Leong, K., & Paige, A. (2022). Implementing an audit and feedback cycle to improve adherence to the Choosing Wisely Canada recommendations: clustered randomized trail. *BMC Primary Care*, *23*(1), 302. | GP-focused |
| 1. Seow, H., Bainbridge, D., Winemaker, S., Stajduhar, K., Pond, G., Kortes-Miller, K., ... & Pereira, J. (2023). Increasing palliative care capacity in primary care: study protocol of a cluster randomized controlled trial of the CAPACITI training program. *BMC Palliative Care*, *22*(1), 2. | GP-focused |
| 1. Arain, M. A., Ahmad, A., Chiu, V., & Kembel, L. (2021). Medication adherence support of an in-home electronic medication dispensing system for individuals living with chronic conditions: a pilot randomized controlled trial. *BMC geriatrics*, *21*, 1-16. | Patient-focused |
| 1. Miklavcic, J. J., Fraser, K. D., Ploeg, J., Markle-Reid, M., Fisher, K., Gafni, A., ... & Upshur, R. (2020). Effectiveness of a community program for older adults with type 2 diabetes and multimorbidity: a pragmatic randomized controlled trial. *Bmc Geriatrics*, *20*, 1-14. | Other |
| 1. McIsaac, W., Kukan, S., Huszti, E., Szadkowski, L., O’Neill, B., Virani, S., ... & Morris, A. M. (2021). A pragmatic randomized trial of a primary care antimicrobial stewardship intervention in Ontario, Canada. *BMC Family Practice*, *22*, 1-8. | GP-focused |
| 1. Singer, A., Kosowan, L., Leong, K., Katz, A., Abrams, E., & Lix, L. (2022). Randomized controlled trial of an audit and feedback cycle to improve adherence to Choosing Wisely Canada recommendations. *Annals of Family* Medicine, *20*(20 Suppl 1), 2883. | GP-focused |
| 1. Fortin, M., Stewart, M., Ngangue, P., Almirall, J., Bélanger, M., Brown, J.B., … & Zwarenstein, M. (2021). Scaling Up Patient-Centered Interdisciplinary Care for Multimorbidity: A Pragmatic Mixed-Methods Randomized Controlled Trial. *Annals of Family* Medicine, *19*(2), 126-134. | Other |

***Patient-focused:** Interventions that are behavioral, educational, or informational, targeting patients. **GP-focused:** Interventions that are behavioral, educational, or informational, targeting general practitioners (GPs). **Other:** Studies evaluating new models of care, assessing the impact of new clinical guidelines, or contributing to research methodology.

**Note:** If multiple papers referred to the same trial (e.g., a study protocol and its corresponding results paper), only one was included. Papers published after 2015 were excluded if their primary findings had already been reported before 2015. **Primary care settings:** Studies were considered to be conducted in primary care if participant recruitment took place in GP practices and/or if the research involved GPs. Studies conducted in secondary care, specialized services (e.g., sexual health clinics, mental health services, physiotherapy, maternity care, nursing homes), pharmacies, or dental practices were excluded. However, exceptions were made for studies comparing models of care, such as those examining primary care versus secondary care. Studies conducted in multiple countries were excluded.

**Table A5. Reference list of randomized controlled trials conducted in primary care settings in Germany, 2015-2025**

| **Reference** | **Intervention focus*** |
| --- | --- |
| 1. Konerding, U., Redaèlli, M., Ackermann, K., Altin, S., Appelbaum, S., Biallas, B., ... & Stock, S. (2021). A pragmatic randomised controlled trial referring to a Personalised Self-management SUPport Programme (P-SUP) for persons enrolled in a disease management programme for type 2 diabetes mellitus and/or for coronary heart disease. *Trials*, *22*, 1-17. | Patient-focused |
| 1. Hammersen, F., Goetz, K., Soennichsen, A., Emcke, T., & Steinhaeuser, J. (2016). Effects of communication training with the MAAS-Global-D instrument on the antibiotic prescribing for respiratory infections in primary care: study protocol of a randomised controlled trial. *Trials*, *17*, 1-9. | GP-focused |
| 1. Härter, M., Bartsch, A. L., Egger, N., König, H. H., Kriston, L., Schulz, H., ... & Buchholz, A. (2015). Evaluating a collaborative smoking cessation intervention in primary care (ENTER): study protocol for a cluster-randomized controlled trial. *Trials*, *16*, 1-9. | Patient-focused |
| 1. Wennehorst, K., Mildenstein, K., Saliger, B., Tigges, C., Diehl, H., Keil, T., & Englert, H. (2016). A comprehensive lifestyle intervention to prevent type 2 diabetes and cardiovascular diseases: the German CHIP trial. *Prevention Science*, *17*(3), 386-397. | Patient-focused |
| 1. Ose, D., Kamradt, M., Kiel, M., Freund, T., Besier, W., Mayer, M., ... & Szecsenyi, J. (2019). Care management intervention to strengthen self-care of multimorbid patients with type 2 diabetes in a German primary care network: A randomized controlled trial. *PloS one*, *14*(6), e0214056. | Other |
| 1. Siebenhofer, A., Ulrich, L. R., Mergenthal, K., Berghold, A., Pregartner, G., Kemperdick, B., ... & Petersen, J. J. (2019). Primary care management for patients receiving long-term antithrombotic treatment: a cluster-randomized controlled trial. *PLoS One*, *14*(1), e0209366. | Other |
| 1. Löwe, B., Scherer, M., Braunschneider, L. E., Marx, G., Eisele, M., Mallon, T., ... & Kohlmann, S. (2024). Clinical effectiveness of patient-targeted feedback following depression screening in general practice (GET. FEEDBACK. GP): an investigator-initiated, prospective, multicentre, three-arm, observer-blinded, randomised controlled trial in Germany. *The Lancet Psychiatry*, *11*(4), 262-273. | Patient-focused |
| 1. Thyrian, J. R., Hertel, J., Wucherer, D., Eichler, T., Michalowsky, B., Dreier-Wolfgramm, A., ... & Hoffmann, W. (2017). Effectiveness and safety of dementia care management in primary care: a randomized clinical trial. *JAMA psychiatry*, *74*(10), 996-1004. | Other |
| 1. Mortsiefer, A., Löscher, S., Pashutina, Y., Santos, S., Altiner, A., Drewelow, E., ... & Feldmeier, G. (2023). Family conferences to facilitate deprescribing in older outpatients with Frailty and with polypharmacy: the COFRAIL Cluster Randomized Trial. *JAMA Network Open*, *6*(3), e234723-e234723. | GP-focused |
| 1. Schmidt, K., Worrack, S., Von Korff, M., Davydow, D., Brunkhorst, F., Ehlert, U., ... & SMOOTH Study Group. (2016). Effect of a primary care management intervention on mental health–related quality of life among survivors of sepsis: a randomized clinical trial. *Jama*, *315*(24), 2703-2711. | Other |
| 1. Koch, R., Rösel, I., Polanc, A., Thies, C., Sundmacher, L., Eigentler, T., ... & Joos, S. (2024). TELEDerm: Implementing store-and-forward teledermatology consultations in general practice: Results of a cluster randomized trial. *Journal of telemedicine and telecare*, *30*(4), 647-660. | Other |
| 1. Haun, M. W., van Eickels, D., Tönnies, J., Graue, L., Ayoub-Schreifeldt, M., Wensing, M., ... & Friederich, H. C. (2024). An integrated mental health video consultations model for patients with somatic symptom disorder in primary care: The randomized VISION pilot trial. *Journal of Psychosomatic Research*, *182*, 111801. | Other |
| 1. Priebe, J. A., Haas, K. K., Moreno Sanchez, L. F., Schoefmann, K., Utpadel-Fischler, D. A., Stockert, P., ... & Toelle, T. R. (2020). Digital treatment of back pain versus standard of care: the cluster-randomized controlled trial, rise-uP. *Journal of pain research*, 1823-1838. | Other |
| 1. Send, A. F. J., Peters‐Klimm, F., Bruckner, T., Haefeli, W. E., & Seidling, H. M. (2017). A randomized controlled trial to assess the effect of a medication plan containing drug administration recommendations on patients’ drug knowledge after 2 months. *Journal of clinical pharmacy and therapeutics*, *42*(1), 69-74. | Patient-focused |
| 1. Zimmermann, T., Puschmann, E., van den Bussche, H., Wiese, B., Ernst, A., Porzelt, S., ... & Scherer, M. (2016). Collaborative nurse-led self-management support for primary care patients with anxiety, depressive or somatic symptoms: Cluster-randomised controlled trial (findings of the SMADS study). *International journal of nursing studies*, *63*, 101-111. | Other |
| 1. Lech, S., Gellert, P., Spang, R. P., Voigt‐Antons, J. N., Huscher, D., O’Sullivan, J. L., & Schuster, J. (2023). Effectiveness of a tablet‐based intervention for people living with dementia in primary care—A cluster randomized controlled trial. *International Journal of Geriatric Psychiatry*, *38*(12), e6035. | Patient-focused |
| 1. Zülke, A. E., Pabst, A., Luppa, M., Roehr, S., Seidling, H., Oey, A., ... & Riedel‐Heller, S. G. (2024). A multidomain intervention against cognitive decline in an at‐risk‐population in Germany: Results from the cluster‐randomized AgeWell. de trial. *Alzheimer's & Dementia*, 20(1), 615-628. | Patient-focused |
| 1. Grochtdreis, T., Brettschneider, C., Bjerregaard, F., Bleich, C., Boczor, S., Härter, M., ... & König, H. H. (2019). Cost-effectiveness analysis of collaborative treatment of late-life depression in primary care (GermanIMPACT). *European Psychiatry*, *57*, 10-18. | Other |
| 1. Brünn, R., Basten, J., Lemke, D., Piotrowski, A., Söling, S., Surmann, B., ... & Muth, C. (2024). Digital Medication Management in Polypharmacy: Findings of a Cluster-Randomized, Controlled Trial With a Stepped-Wedge Design in Primary Care Practices (AdAM). *Deutsches Ärzteblatt International*, *121*(8), 243. | GP-focused |
| 1. Rudolf, H., Thiem, U., Aust, K., Krause, D., Klaaßen-Mielke, R., Greiner, W., ... & Wilm, S. (2021). Reduction of potentially inappropriate medication in the elderly: Results of a cluster-randomized, controlled trial in German primary care practices (RIME). *Deutsches Ärzteblatt International*, *118*(51-52), 875. | GP-focused |
| 1. Hölzel, L. P., Bjerregaard, F., Bleich, C., Boczor, S., Härter, M., König, H. H., ... & Hüll, M. (2018). Coordinated Treatment of Depression in Elderly People in Primary Care: A Cluster-Randomized, Controlled Study (GermanIMPACT). *Deutsches Ärzteblatt International*, *115*(44), 741. | Other |
| 1. Gágyor, I., Hummers, E., Schmiemann, G., Friede, T., Pfeiffer, S., Afshar, K., & Bleidorn, J. (2021). Herbal treatment with uva ursi extract versus fosfomycin in women with uncomplicated urinary tract infection in primary care: a randomized controlled trial. *Clinical Microbiology and Infection*, *27*(10), 1441-1447. | Other |
| 1. Muth, C., Uhlmann, L., Haefeli, W. E., Rochon, J., van den Akker, M., Perera, R., ... & Harder, S. (2018). Effectiveness of a complex intervention on Prioritising Multimedication in Multimorbidity (PRIMUM) in primary care: results of a pragmatic cluster randomised controlled trial. *BMJ open*, *8*(2), e017740. | GP-focused |
| 1. Schäfer, I., Kaduszkiewicz, H., Mellert, C., Löffler, C., Mortsiefer, A., Ernst, A., ... & Altiner, A. (2018). Narrative medicine-based intervention in primary care to reduce polypharmacy: results from the cluster-randomised controlled trial MultiCare AGENDA. *BMJ open*, *8*(1), e017653. | Patient-focused |
| 1. Haun, M. W., Tönnies, J., Hartmann, M., Wildenauer, A., Wensing, M., Szecsenyi, J., ... & Friederich, H. C. (2024). Model of integrated mental health video consultations for people with depression or anxiety in primary care (PROVIDE-C): assessor masked, multicentre, randomised controlled trial. *bmj*, *386*. | Other |
| 1. Schmiemann, G., Greser, A., Maun, A., Bleidorn, J., Schuster, A., Miljukov, O., ... & Gágyor, I. (2023). Effects of a multimodal intervention in primary care to reduce second line antibiotic prescriptions for urinary tract infections in women: parallel, cluster randomised, controlled trial. *bmj*, *383*. | GP-focused |
| 1. Wächtler, H., Kaduszkiewicz, H., Kuhnert, O., Malottki, K. A., Maaß, S., Hedderich, J., ... & Hansmann-Wiest, J. (2023). Influence of a guideline or an additional rapid strep test on antibiotic prescriptions for sore throat: the cluster randomized controlled trial of HALS (Hals und Antibiotika Leitlinien Strategien). *BMC Primary Care*, *24*(1), 75. | GP-focused |
| 1. Wegwarth, O., Spies, C., Ludwig, W. D., Donner-Banzhoff, N., Jonitz, G., & Hertwig, R. (2022). Educating physicians on strong opioids by descriptive versus simulated-experience formats: a randomized controlled trial. *BMC medical education*, *22*(1), 741. | GP-focused |
| 1. Maikranz, V., Siebenhofer, A., Ulrich, L. R., Mergenthal, K., Schulz-Rothe, S., Kemperdick, B., ... & Petersen, J. J. (2017). Does a complex intervention increase patient knowledge about oral anticoagulation?-a cluster-randomised controlled trial. *BMC family practice*, *18*, 1-10. | Patient-focused |
| 1. Rose, O., Schaffert, C., Czarnecki, K., Mennemann, H. S., Waltering, I., Hamacher, S., ... & Köberlein, J. (2015). Effect evaluation of an interprofessional medication therapy management approach for multimorbid patients in primary care: a cluster-randomized controlled trial in community care (WestGem study protocol). *BMC family practice*, *16*, 1-11. | Other |
| 1. Seidel-Jacobs, E., Kohl, F., Rosenbauer, J., Schulze, M. B., Kuss, O., & Rathmann, W. (2025). Effect of applying a diabetes risk score on lifestyle counselling and shared decision-making in primary care: A pragmatic cluster randomised trial. *Primary Care Diabetes*, 19(1), 86-91. | Patient-focused |
| 1. Freund, T., Peters-Klimm, F., Boyd, C. M., Mahler, C., Gensichen, J., Erler, A., ... & Szecsenyi, J. (2016). Medical assistant–based care management for high-risk patients in small primary care practices: a cluster randomized clinical trial. *Annals of internal medicine*, *164*(5), 323-330 | Other |
| 1. Mallon, T., Schulze, J., Dams, J., Weber, J., Asendorf, T., Böttcher, S., ... & Marx, G. (2024). Evaluating palliative care case conferences in primary care for patients with advanced non-malignant chronic conditions: a cluster-randomised controlled trial (KOPAL). *Age and Ageing*, *53*(5), afae100. | Other |

***Patient-focused:** Interventions that are behavioral, educational, or informational, targeting patients. **GP-focused:** Interventions that are behavioral, educational, or informational, targeting general practitioners (GPs). **Other:** Studies evaluating new models of care, assessing the impact of new clinical guidelines, or contributing to research methodology.

**Note:** If multiple papers referred to the same trial (e.g., a study protocol and its corresponding results paper), only one was included. Papers published after 2015 were excluded if their primary findings had already been reported before 2015. **Primary care settings:** Studies were considered to be conducted in primary care if participant recruitment took place in GP practices and/or if the research involved GPs. Studies conducted in secondary care, specialized services (e.g., sexual health clinics, mental health services, physiotherapy, maternity care, nursing homes), pharmacies, or dental practices were excluded. However, exceptions were made for studies comparing models of care, such as those examining primary care versus secondary care. Studies conducted in multiple countries were excluded.

**Table A6. Reference list of randomized controlled trials conducted in primary care settings in the UK, 2015-2025**

| **Reference** | **Intervention focus*** |
| --- | --- |
| 1. Burton, C., Mooney, C., Sutton, L., White, D., Dawson, J., Neilson, A. R., ... & Deary, V. (2024). Effectiveness of a symptom-clinic intervention delivered by general practitioners with an extended role for people with multiple and persistent physical symptoms in England: the Multiple Symptoms Study 3 pragmatic, multicentre, parallel-group, individually randomised controlled trial. *The Lancet*, *403*(10444), 2619-2629. | Patient-focused |
| 1. Hawkey, C., Avery, A., Coupland, C. A., Crooks, C., Dumbleton, J., Hobbs, F. R., ... & O'Brien, W. (2022). Helicobacter pylori eradication for primary prevention of peptic ulcer bleeding in older patients prescribed aspirin in primary care (HEAT): a randomised, double-blind, placebo-controlled trial. *The Lancet*, *400*(10363), 1597-1606. | Other |
| 1. Sugg, H. V. R., Frost, J., & Richards, D. A. (2020). Personalising psychotherapies for depression using a novel mixed methods approach: an example from Morita therapy. *Trials*, *21*, 1-12. | Patient-focused |
| 1. Cotterill, S., Howells, K., Rhodes, S., & Bower, P. (2017). The effect of using social pressure in cover letters to improve retention in a longitudinal health study: an embedded randomised controlled retention trial. *Trials*, *18*, 1-9. | Patient-focused |
| 1. Flower, A., Harman, K., Lewith, G., Moore, M., Bishop, F. L., Stuart, B., & Lampert, N. (2016). Standardised Chinese herbal treatment delivered by GPs compared with individualised treatment administered by practitioners of Chinese herbal medicine for women with recurrent urinary tract infections (RUTI): Study protocol for a randomised controlled trial. *Trials*, *17*, 1-9. | Other |
| 1. Osborn, D., Burton, A., Walters, K., Nazareth, I., Heinkel, S., Atkins, L., ... & Robinson, V. (2016). Evaluating the clinical and cost effectiveness of a behaviour change intervention for lowering cardiovascular disease risk for people with severe mental illnesses in primary care (PRIMROSE study): study protocol for a cluster randomised controlled trial. *Trials*, *17*, 1-12. | Patient-focused |
| 1. Ridd, M. J., Garfield, K., Gaunt, D. M., Hollinghurst, S., Redmond, N. M., Powell, K., ... & Metcalfe, C. (2016). Choice of Moisturiser for Eczema Treatment (COMET): feasibility study of a randomised controlled parallel group trial in children recruited from primary care. *BMJ open*, 6(11), e012021. | Other |
| 1. Yates, T., Griffin, S., Bodicoat, D. H., Brierly, G., Dallosso, H., Davies, M. J., ... & Khunti, K. (2015). PRomotion Of Physical activity through structured Education with differing Levels of ongoing Support for people at high risk of type 2 diabetes (PROPELS): study protocol for a randomized controlled trial. *Trials*, *16*, 1-16. | Patient-focused |
| 1. Martin, K. R., Stelfox, K., Macfarlane, G. J., McNamee, P., Morrison, Z., & Smith, T. O. (2023). Bringing the Walk with Ease Programme to the UK: a mixed-methods study to assess the relevance, acceptability, and feasibility of implementation for people with arthritis and musculoskeletal conditions. *Translational behavioral medicine*, *13*(11), 851-866. | Patient-focused |
| 1. Hill, J. C., Garvin, S., Bromley, K., Saunders, B., Kigozi, J., Cooper, V., ... & Foster, N. E. (2022). Risk-based stratified primary care for common musculoskeletal pain presentations (STarT MSK): a cluster-randomised, controlled trial. *The Lancet Rheumatology*, *4*(9), e591-e602. | Other |
| 1. Kassavou, A., Mirzaei, V., Shpendi, S., Brimicombe, J., Chauhan, J., Bhattacharya, D., ... & Sutton, S. (2021). The feasibility of the PAM intervention to support treatment-adherence in people with hypertension in primary care: a randomised clinical controlled trial. *Scientific Reports*, *11*(1), 8897. | Patient-focused |
| 1. Lewis, G., Duffy, L., Ades, A., Amos, R., Araya, R., Brabyn, S., ... & Lewis, G. (2019). The clinical effectiveness of sertraline in primary care and the role of depression severity and duration (PANDA): a pragmatic, double-blind, placebo-controlled randomised trial. *The Lancet Psychiatry*, *6*(11), 903-914. | Other |
| 1. Dennick, K., Bridle, C., & Sturt, J. (2015). Written emotional disclosure for adults with Type 2 diabetes: a primary care feasibility study. *Primary health care research & development*, *16*(2), 179-187. | Patient-focused |
| 1. Kaushal, A., Hirst, Y., Tookey, S., Kerrison, R. S., Marshall, S., Prentice, A., ... & von Wagner, C. (2020). Use of a GP-endorsed non-participant reminder letter to promote uptake of bowel scope screening: A randomised controlled trial in a hard-to-reach population. *Preventive medicine*, *141*, 106268. | Patient-focused |
| 1. Davies, M. J., Gray, L. J., Troughton, J., Gray, A., Tuomilehto, J., Farooqi, A., ... & Yates, T. (2016). A community based primary prevention programme for type 2 diabetes integrating identification and lifestyle intervention for prevention: the Let's Prevent Diabetes cluster randomised controlled trial. *Preventive medicine*, *84*, 48-56. | Patient-focused |
| 1. El-Gohary, M., Moore, M., Roderick, P., Watkins, E., Dash, J., Reinson, T., ... & Sheron, N. (2018). Local care and treatment of liver disease (LOCATE)–a cluster-randomized feasibility study to discover, assess and manage early liver disease in primary care. *PLoS One*, *13*(12), e0208798. | Other |
| 1. Griffiths, C., Bremner, S., Islam, K., Sohanpal, R., Vidal, D. L., Dawson, C., ... & Eldridge, S. (2016). Effect of an education programme for South Asians with asthma and their clinicians: a cluster randomised controlled trial (OEDIPUS). *PLoS One*, *11*(12), e0158783. | Patient-focused |
| 1. Piernas, C., Aveyard, P., Lee, C., Tsiountsioura, M., Noreik, M., Astbury, N. M., ... & Jebb, S. A. (2020). Evaluation of an intervention to provide brief support and personalized feedback on food shopping to reduce saturated fat intake (PC-SHOP): A randomized controlled trial. *PLoS Medicine*, *17*(11), e1003385. | Patient-focused |
| 1. Harris, T., Kerry, S. M., Limb, E. S., Victor, C. R., Iliffe, S., Ussher, M., ... & Cook, D. G. (2017). Effect of a Primary Care Walking Intervention with and without Nurse Support on Physical Activity Levels in 45-to 75-Year-Olds: The Pedometer And Consultation Evaluation (PACE-UP) Cluster Randomised Clinical Trial. *PLoS medicine*, *14*(1), e1002210. | Patient-focused |
| 1. Livingston, G., Baio, G., Sommerlad, A., de Lusignan, S., Poulimenos, S., Morris, S., ... & Hoe, J. (2017). Effectiveness of an intervention to facilitate prompt referral to memory clinics in the United Kingdom: cluster randomised controlled trial. *PLoS Medicine*, *14*(3), e1002252. | Patient-focused |
| 1. Godino, J. G., Van Sluijs, E. M., Marteau, T. M., Sutton, S., Sharp, S. J., & Griffin, S. J. (2016). Lifestyle advice combined with personalized estimates of genetic or phenotypic risk of type 2 diabetes, and objectively measured physical activity: a randomized controlled trial. *PLoS medicine*, *13*(11), e1002185. | Patient-focused |
| 1. Harris, T., Kerry, S. M., Victor, C. R., Ekelund, U., Woodcock, A., Iliffe, S., ... & Cook, D. G. (2015). A primary care nurse-delivered walking intervention in older adults: PACE (pedometer accelerometer consultation evaluation)-Lift cluster randomised controlled trial. *PLoS medicine*, *12*(2), e1001783. | Patient-focused |
| 1. Bishop, A., Ogollah, R. O., Jowett, S., Kigozi, J., Tooth, S., Protheroe, J., ... & Foster, N. E. (2017). STEMS pilot trial: a pilot cluster randomised controlled trial to investigate the addition of patient direct access to physiotherapy to usual GP-led primary care for adults with musculoskeletal pain. *BMJ open*, *7*(3), e012987. | Other |
| 1. Leydon, G. M., Stuart, B., Summers, R. H., Little, P., Ekberg, S., Stevenson, F., ... & Moore, M. V. (2018). Findings from a feasibility study to improve GP elicitation of patient concerns in UK general practice consultations. *Patient Education and Counseling*, *101*(8), 1394-1402. | GP-focused |
| 1. Shepstone, L., Lenaghan, E., Cooper, C., Clarke, S., Fong-Soe-Khioe, R., Fordham, R., ... & Mitchell, N. (2018). Screening in the community to reduce fractures in older women (SCOOP): a randomised controlled trial. *The Lancet*, *391*(10122), 741-747. | Other |
| 1. Hin, H., Tomson, J., Newman, C., Kurien, R., Lay, M., Cox, J., ... & Clarke, R. (2017). Optimum dose of vitamin D for disease prevention in older people: BEST-D trial of vitamin D in primary care. *Osteoporosis International*, *28*, 841-851. | Other |
| 1. Dziedzic, K. S., Healey, E. L., Porcheret, M., Afolabi, E. K., Lewis, M., Morden, A., ... & Hay, E. M. (2018). Implementing core NICE guidelines for osteoarthritis in primary care with a model consultation (MOSAICS): a cluster randomised controlled trial. *Osteoarthritis and cartilage*, *26*(1), 43-53. | Other |
| 1. Dallosso, H., Mandalia, P., Gray, L. J., Chudasama, Y. V., Choudhury, S., Taheri, S., ... & Davies, M. J. (2022). The effectiveness of a structured group education programme for people with established type 2 diabetes in a multi-ethnic population in primary care: A cluster randomised trial. *Nutrition, Metabolism and Cardiovascular Diseases*, *32*(6), 1549-1559. | Patient-focused |
| 1. Lewis, G., Marston, L., Duffy, L., Freemantle, N., Gilbody, S., Hunter, R., ... & Lewis, G. (2021). Maintenance or discontinuation of antidepressants in primary care. *New England Journal of Medicine*, *385*(14), 1257-1267. | Other |
| 1. Geraghty, A. W., Becque, T., Roberts, L. C., Hill, J. C., Foster, N. E., Yardley, L., ... & Little, P. (2024). Supporting self-management of low back pain with an internet intervention with and without telephone support in primary care (SupportBack 2): a randomised controlled trial of clinical and cost-effectiveness. *The Lancet Rheumatology*. | Patient-focused |
| 1. Little, P., Vennik, J., Rumsby, K., Stuart, B., Becque, T., Moore, M., ... & Geraghty, A. W. (2024). Nasal sprays and behavioural interventions compared with usual care for acute respiratory illness in primary care: a randomised, controlled, open-label, parallel-group trial. *The Lancet Respiratory Medicine*, *12*(8), 619-632. | Patient-focused |
| 1. Ramakrishnan, S., Jeffers, H., Langford-Wiley, B., Davies, J., Thulborn, S. J., Mahdi, M., ... & Bafadhel, M. (2024). Blood eosinophil-guided oral prednisolone for COPD exacerbations in primary care in the UK (STARR2): a non-inferiority, multicentre, double-blind, placebo-controlled, randomised controlled trial. *The Lancet Respiratory Medicine*, *12*(1), 67-77. | Other |
| 1. Butler, C. C., Yu, L. M., Dorward, J., Gbinigie, O., Hayward, G., Saville, B. R., ... & Hobbs, F. R. (2021). Doxycycline for community treatment of suspected COVID-19 in people at high risk of adverse outcomes in the UK (PRINCIPLE): a randomised, controlled, open-label, adaptive platform trial. *The Lancet Respiratory Medicine*, *9*(9), 1010-1020. | Other |
| 1. Bruton, A., Lee, A., Yardley, L., Raftery, J., Arden-Close, E., Kirby, S., ... & Thomas, M. (2018). Physiotherapy breathing retraining for asthma: a randomised controlled trial. *The Lancet Respiratory Medicine*, *6*(1), 19-28. | Patient-focused |
| 1. Jordan, R. E., Adab, P., Sitch, A., Enocson, A., Blissett, D., Jowett, S., ... & Fitzmaurice, D. (2016). Targeted case finding for chronic obstructive pulmonary disease versus routine practice in primary care (TargetCOPD): a cluster-randomised controlled trial. *The Lancet Respiratory Medicine*, *4*(9), 720-730. | Other |
| 1. Ahern, A. L., Breeze, P., Fusco, F., Sharp, S. J., Islam, N., Wheeler, G. M., ... & Griffin, S. J. (2022). Effectiveness and cost-effectiveness of referral to a commercial open group behavioural weight management programme in adults with overweight and obesity: 5-year follow-up of the WRAP randomised controlled trial. *The Lancet Public Health*, *7*(10), e866-e875. | Patient-focused |
| 1. Morriss, R., Patel, S., Boutry, C., Patel, P., Guo, B., Briley, P. M., ... & Kai, J. (2023). Clinical effectiveness of active Alpha-Stim AID versus sham Alpha-Stim AID in major depression in primary care in England (Alpha-Stim-D): a multicentre, parallel group, double-blind, randomised controlled trial. *The Lancet Psychiatry*, *10*(3), 172-183. | Other |
| 1. Wiles, N. J., Thomas, L., Turner, N., Garfield, K., Kounali, D., Campbell, J., ... & Hollinghurst, S. (2016). Long-term effectiveness and cost-effectiveness of cognitive behavioural therapy as an adjunct to pharmacotherapy for treatment-resistant depression in primary care: follow-up of the CoBalT randomised controlled trial. *The Lancet Psychiatry*, *3*(2), 137-144. | Patient-focused |
| 1. Perez, J., Jin, H., Russo, D. A., Stochl, J., Painter, M., Shelley, G., ... & Jones, P. B. (2015). Clinical effectiveness and cost-effectiveness of tailored intensive liaison between primary and secondary care to identify individuals at risk of a first psychotic illness (the LEGs study): a cluster-randomised controlled trial. *The Lancet Psychiatry*, *2*(11), 984-993. | Other |
| 1. Leber, W., McMullen, H., Anderson, J., Marlin, N., Santos, A. C., Bremner, S., ... & Griffiths, C. (2015). Promotion of rapid testing for HIV in primary care (RHIVA2): a cluster-randomised controlled trial. *The lancet HIV*, *2*(6), e229-e235. | Other |
| 1. Gilbody, S., Littlewood, E., McMillan, D., Atha, L., Bailey, D., Baird, K., ... & Ekers, D. (2024). Behavioural activation to mitigate the psychological impacts of COVID-19 restrictions on older people in England and Wales (BASIL+): a pragmatic randomised controlled trial. *The Lancet Healthy Longevity*, *5*(2), e97-e107. | Patient-focused |
| 1. Flanagan, S., Kunkel, J., Appleby, V., Eldridge, S. E., Ismail, S., Moreea, S., ... & Foster, G. R. (2019). Case finding and therapy for chronic viral hepatitis in primary care (HepFREE): a cluster-randomised controlled trial. *The Lancet Gastroenterology & Hepatology*, *4*(1), 32-44. | GP-focused |
| 1. Lean, M. E., Leslie, W. S., Barnes, A. C., Brosnahan, N., Thom, G., McCombie, L., ... & Taylor, R. (2018). Primary care-led weight management for remission of type 2 diabetes (DiRECT): an open-label, cluster-randomised trial. *The Lancet*, *391*(10120), 541-551. | Patient-focused |
| 1. Ford, A. C., Wright-Hughes, A., Alderson, S. L., Ow, P. L., Ridd, M. J., Foy, R., ... & Everitt, H. A. (2023). Amitriptyline at low-dose and titrated for irritable bowel syndrome as second-line treatment in primary care (ATLANTIS): a randomised, double-blind, placebo-controlled, phase 3 trial. *The Lancet*, *402*(10414), 1773-1785. | Other |
| 1. Kyle, S. D., Siriwardena, A. N., Espie, C. A., Yang, Y., Petrou, S., Ogburn, E., ... & Aveyard, P. (2023). Clinical and cost-effectiveness of nurse-delivered sleep restriction therapy for insomnia in primary care (HABIT): a pragmatic, superiority, open-label, randomised controlled trial. *The Lancet*, *402*(10406), 975-987. | Patient-focused |
| 1. Mackenzie, I. S., Hawkey, C. J., Ford, I., Greenlaw, N., Pigazzani, F., Rogers, A., ... & Zutis, K. (2022). Allopurinol versus usual care in UK patients with ischaemic heart disease (ALL-HEART): a multicentre, prospective, randomised, open-label, blinded-endpoint trial. *The Lancet*, *400*(10359), 1195-1205. | Other |
| 1. Fitzgerald, R. C., di Pietro, M., O'Donovan, M., Maroni, R., Muldrew, B., Debiram-Beecham, I., ... & Hunt, M. (2020). Cytosponge-trefoil factor 3 versus usual care to identify Barrett's oesophagus in a primary care setting: a multicentre, pragmatic, randomised controlled trial. *The Lancet*, *396*(10247), 333-344. | Other |
| 1. Doherty, M., Jenkins, W., Richardson, H., Sarmanova, A., Abhishek, A., Ashton, D., ... & Zhang, W. (2018). Efficacy and cost-effectiveness of nurse-led care involving education and engagement of patients and a treat-to-target urate-lowering strategy versus usual care for gout: a randomised controlled trial. *The Lancet*, *392*(10156), 1403-1412. | Patient-focused |
| 1. McManus, R. J., Mant, J., Franssen, M., Nickless, A., Schwartz, C., Hodgkinson, J., ... & Cubitt, C. (2018). Efficacy of self-monitored blood pressure, with or without telemonitoring, for titration of antihypertensive medication (TASMINH4): an unmasked randomised controlled trial. *The Lancet*, *391*(10124), 949-959. | Patient-focused |
| 1. Woodcock, A., Vestbo, J., Bakerly, N. D., New, J., Gibson, J. M., McCorkindale, S., ... & Wright, A. T. (2017). Effectiveness of fluticasone furoate plus vilanterol on asthma control in clinical practice: an open-label, parallel group, randomised controlled trial. *The Lancet*, *390*(10109), 2247-2255. | Other |
| 1. Kuyken, W., Hayes, R., Barrett, B., Byng, R., Dalgleish, T., Kessler, D., ... & Byford, S. (2015). Effectiveness and cost-effectiveness of mindfulness-based cognitive therapy compared with maintenance antidepressant treatment in the prevention of depressive relapse or recurrence (PREVENT): a randomised controlled trial. *The Lancet*, *386*(9988), 63-73. | Patient-focused |
| 1. Riches, S. P., Piernas, C., Aveyard, P., Sheppard, J. P., Rayner, M., Albury, C., & Jebb, S. A. (2021). A mobile health salt reduction intervention for people with hypertension: results of a feasibility randomized controlled trial. *JMIR mHealth and uHealth*, *9*(10), e26233. | Patient-focused |
| 1. Kendrick, T., Stuart, B., Bowers, H., Sadeghi, M. H., Page, H., Dowrick, C., ... & Geraghty, A. W. (2024). Internet and telephone support for discontinuing long-term antidepressants: the REDUCE cluster randomized trial. *JAMA network open*, *7*(6), e2418383-e2418383. | GP-focused |
| 1. Walter, F. M., Pannebakker, M. M., Barclay, M. E., Mills, K., Saunders, C. L., Murchie, P., ... & Emery, J. D. (2020). Effect of a skin self-monitoring smartphone application on time to physician consultation among patients with possible melanoma: a phase 2 randomized clinical trial. *JAMA network open*, *3*(2), e200001-e200001. | Patient-focused |
| 1. Hayward, G., Mort, S., Hay, A. D., Moore, M., Thomas, N. P., Cook, J., ... & Butler, C. C. (2024). d-Mannose for Prevention of Recurrent Urinary Tract Infection Among Women: A Randomized Clinical Trial. *JAMA Internal Medicine*, *184*(6), 619-628. | Other |
| 1. Martin, R. M., Donovan, J. L., Turner, E. L., Metcalfe, C., Young, G. J., Walsh, E. I., ... & CAP Trial Group. (2018). Effect of a low-intensity PSA-based screening intervention on prostate cancer mortality: the CAP randomized clinical trial. *Jama*, *319*(9), 883-895. | Patient-focused |
| 1. Gilbody, S., Lewis, H., Adamson, J., Atherton, K., Bailey, D., Birtwistle, J., ... & McMillan, D. (2017). Effect of collaborative care vs usual care on depressive symptoms in older adults with subthreshold depression: the CASPER randomized clinical trial. *Jama*, *317*(7), 728-737 | Other |
| 1. McHugh, N., Tillett, W., Helliwell, P., Packham, J., Collier, H., Davies, C., ... & Brown, S. T. (2024). Enhanced surveillance for the detection of psoriatic arthritis in a UK primary care psoriasis population: results from the TUDOR trial. *Rheumatology*, keae374. | Other |
| 1. Kassavou, A., Mirzaei, V., Brimicombe, J., Edwards, S., Massou, E., Prevost, A. T., ... & Sutton, S. (2020). A highly tailored text and voice messaging intervention to improve medication adherence in patients with either or both hypertension and type 2 diabetes in a UK primary care setting: feasibility randomized controlled trial of clinical effectiveness. *Journal of medical Internet research*, *22*(5), e16629 | Patient-focused |
| 1. Bishop, F. L., Greville-Harris, M., Bostock, J., Din, A., Graham, C. A., Lewith, G., ... & Yardley, L. (2019). Informing adults with back pain about placebo effects: randomized controlled evaluation of a new website with potential to improve informed consent in clinical research. *Journal of Medical Internet Research*, *21*(1), e9955. | Patient-focused |
| 1. Murray, E., Sweeting, M., Dack, C., Pal, K., Modrow, K., Hudda, M., ... & Patterson, D. (2017). Web-based self-management support for people with type 2 diabetes (HeLP-Diabetes): randomised controlled trial in English primary care. *BMJ open*, *7*(9), e016009. | Patient-focused |
| 1. Hill, N. R., Groves, L., Dickerson, C., Ochs, A., Pang, D., Lawton, S., ... & Cohen, A. T. (2022). Identification of undiagnosed atrial fibrillation using a machine learning risk-prediction algorithm and diagnostic testing (PULsE-AI) in primary care: a multi-centre randomized controlled trial in England. *European Heart Journal-Digital Health*, *3*(2), 195-204. | Patient-focused |
| 1. Ainsworth, B., Stanescu, S., Stuart, B., Russell, D., Liddiard, M., Djukanovic, R., & Thomas, M. (2022). A feasibility trial of a digital mindfulness-based intervention to improve asthma-related quality of life for primary care patients with asthma. *Journal of Behavioral Medicine*, *45*(1), 133-147. | Patient-focused |
| 1. Marwick, C. A., Hossain, A., Nogueira, R., Sneddon, J., Kavanagh, K., Bennie, M., ... & Malcolm, W. (2022). Feedback of Antibiotic Prescribing in Primary Care (FAPPC) trial: results of a real-world cluster randomized controlled trial in Scotland, UK. *Journal of Antimicrobial Chemotherapy*, *77*(12), 3291-3300. | GP-focused |
| 1. Major, R. W., Brown, C., Shepherd, D., Rogers, S., Pickering, W., Warwick, G. L., ... & Brunskill, N. J. (2019). The primary-secondary care partnership to improve outcomes in chronic kidney disease (PSP-CKD) study: a cluster randomized trial in primary care. *Journal of the American Society of Nephrology*, *30*(7), 1261-1270. | Other |
| 1. Witham, M. D., Price, R. J., Band, M. M., Hannah, M. S., Fulton, R. L., Clarke, C. L., ... & Soiza, R. L. (2019). Effect of vitamin K2 on postural sway in older people who fall: a randomized controlled trial. *Journal of the American Geriatrics Society*, *67*(10), 2102-2107. | Other |
| 1. Peacock, O. J., Western, M. J., Batterham, A. M., Chowdhury, E. A., Stathi, A., Standage, M., ... & Thompson, D. (2020). Effect of novel technology-enabled multidimensional physical activity feedback in primary care patients at risk of chronic disease–the MIPACT study: a randomised controlled trial. *International Journal of Behavioral Nutrition and Physical Activity*, *17*, 1-13. | Patient-focused |
| 1. Smith, J. R., Greaves, C. J., Thompson, J. L., Taylor, R. S., Jones, M., Armstrong, R., ... & Abraham, C. (2019). The community-based prevention of diabetes (ComPoD) study: a randomised, waiting list controlled trial of a voluntary sector-led diabetes prevention programme. *International Journal of Behavioral Nutrition and Physical Activity*, *16*, 1-14. | Patient-focused |
| 1. Beeken, R. J., Leurent, B., Vickerstaff, V., Wilson, R., Croker, H., Morris, S., ... & Wardle, J. (2017). A brief intervention for weight control based on habit-formation theory delivered through primary care: results from a randomised controlled trial. *International Journal of Obesity*, *41*(2), 246-254. | Patient-focused |
| 1. Greaves, C., Gillison, F., Stathi, A., Bennett, P., Reddy, P., Dunbar, J., ... & Taylor, G. (2015). Waste the waist: a pilot randomised controlled trial of a primary care based intervention to support lifestyle change in people with high cardiovascular risk. *International Journal of Behavioral Nutrition and Physical Activity*, *12*, 1-13. | Patient-focused |
| 1. Ainsworth, B., Horwood, J., Walter, S. R., Miller, S., Chalder, M., De Vocht, F., ... & Yardley, L. (2023). Implementing Germ Defence digital behaviour change intervention via all primary care practices in England to reduce respiratory infections during the COVID-19 pandemic: an efficient cluster randomised controlled trial using the OpenSAFELY platform. *Implementation Science*, *18*(1), 67. | Patient-focused |
| 1. Presseau, J., Mackintosh, J., Hawthorne, G., Francis, J. J., Johnston, M., Grimshaw, J. M., ... & Sniehotta, F. F. (2018). Cluster randomised controlled trial of a theory-based multiple behaviour change intervention aimed at healthcare professionals to improve their management of type 2 diabetes in primary care. *Implementation Science*, *13*, 1-10. | GP-focused |
| 1. Kendrick, T., Dowrick, C., Lewis, G., Moore, M., Leydon, G. M., Geraghty, A. W., ... & Stuart, B. (2024). Patient-reported outcome measures for monitoring primary care patients with depression: the PROMDEP cluster RCT and economic evaluation. *Health Technology Assessment (Winchester, England)*, *28*(17), 1. | Other |
| 1. Worthington, J., Frost, J., Sanderson, E., Cochrane, M., Wheeler, J., Cotterill, N., ... & TRIUMPH study group. (2024). Lower urinary tract symptoms in men: the TRIUMPH cluster RCT. *Health Technology Assessment (Winchester, England)*, *28*(13), 1. | Other |
| 1. Blair, P. S., Young, G. J., Clement, C., Dixon, P., Seume, P., Ingram, J., ... & Hay, A. D. (2023). A multifaceted intervention to reduce antibiotic prescribing among CHIldren with acute COugh and respiratory tract infection: the CHICO cluster RCT. *Health Technology Assessment (Winchester, England)*, *27*(32), 1. | GP-focused |
| 1. Ridd, M. J., Wells, S., MacNeill, S. J., Sanderson, E., Webb, D., Banks, J., ... & Santer, M. (2023). Comparison of lotions, creams, gels and ointments for the treatment of childhood eczema: the BEE RCT. *Health Technology Assessment (Winchester, England)*, *27*(19), 1. | Other |
| 1. Duffy, L., Clarke, C. S., Lewis, G., Marston, L., Freemantle, N., Gilbody, S., ... & Pervin, J. (2021). Antidepressant medication to prevent depression relapse in primary care: the ANTLER RCT. *Health Technology Assessment*, *25*(69). | Other |
| 1. Bruce, J., Hossain, A., Lall, R., Withers, E. J., Finnegan, S., Underwood, M., ... & Lamb, S. E. (2021). Fall prevention interventions in primary care to reduce fractures and falls in people aged 70 years and over: the PreFIT three-arm cluster RCT. *Health Technology Assessment (Winchester, England)*, *25*(34), 1. | Other |
| 1. Herrett, E., Williamson, E., Brack, K., Perkins, A., Thayne, A., Shakur-Still, H., ... & Smeeth, L. (2021). The effect of statins on muscle symptoms in primary care: the StatinWISE series of 200 N-of-1 RCTs. *Health Technology Assessment (Winchester, England)*, *25*(16), 1. | Other |
| 1. Foster, N. E., Konstantinou, K., Lewis, M., Ogollah, R., Saunders, B., Kigozi, J., ... & Dunn, K. M. (2020). Stratified versus usual care for the management of primary care patients with sciatica: the SCOPiC RCT. *Health Technology Assessment (Winchester, England)*, *24*(49), 1. | Other |
| 1. Francis, N. A., Gillespie, D., White, P., Bates, J., Lowe, R., Sewell, B., ... & Butler, C. C. (2020). C-reactive protein point-of-care testing for safely reducing antibiotics for acute exacerbations of chronic obstructive pulmonary disease: the PACE RCT. *Health Technology Assessment (Winchester, England)*, *24*(15), 1. | Other |
| 1. Hay, A. D., Downing, H., Francis, N. A., Young, G. J., Clement, C., Harris, S. D., ... & Moore, M. V. (2019). Anaesthetic-analgesic ear drops to reduce antibiotic consumption in children with acute otitis media: the CEDAR RCT. *Health Technology Assessment (Winchester, England)*, *23*(34), 1. | Other |
| 1. Gulliford, M. C., Juszczyk, D., Prevost, A. T., Soames, J., McDermott, L., Sultana, K., ... & Charlton, J. (2019). Electronically delivered interventions to reduce antibiotic prescribing for respiratory infections in primary care: cluster RCT using electronic health records and cohort study. *Health technology assessment (Winchester, England)*, *23*(11), 1. | GP-focused |
| 1. Santer, M., Rumsby, K., Ridd, M. J., Francis, N. A., Stuart, B., Chorozoglou, M., ... & Little, P. (2018). Adding emollient bath additives to standard eczema management for children with eczema: the BATHE RCT. *Health Technology Assessment*, *22*(57), 1-116. | Other |
| 1. Bosanquet, K., Adamson, J., Atherton, K., Bailey, D., Baxter, C., Beresford-Dent, J., ... & Gilbody, S. (2017). CollAborative care for Screen-Positive EldeRs with major depression (CASPER plus): a multicentred randomised controlled trial of clinical effectiveness and cost-effectiveness. *Health Technology Assessment (Winchester, England)*, *21*(67), 1. | Other |
| 1. Thomas, M., Bruton, A., Little, P., Holgate, S., Lee, A., Yardley, L., ... & Taylor, L. (2017). A randomised controlled study of the effectiveness of breathing retraining exercises taught by a physiotherapist either by instructional DVD or in face-to-face sessions in the management of asthma in adults. *Health Technology Assessment*, *21*(53), 1-161 | Patient-focused |
| 1. Gabbay, M. B., Ring, A., Byng, R., Anderson, P., Taylor, R. S., Matthews, C., ... & Warner, M. (2017). Debt Counselling for Depression in Primary Care: an adaptive randomised controlled pilot trial (DeCoDer study). *Health Technology Assessment (Winchester, England)*, *21*(35), 1. | Patient-focused |
| 1. Little, P., Stuart, B., Hobbs, F. R., Kelly, J., Smith, E. R., Bradbury, K. J., ... & Yardley, L. (2017). Randomised controlled trial and economic analysis of an internet-based weight management programme: POWeR+ (Positive Online Weight Reduction). *Health technology assessment*, *21*(4). | Patient-focused |
| 1. McRobbie, H., Hajek, P., Peerbux, S., Kahan, B. C., Eldridge, S., Trépel, D., ... & Smith, K. M. (2016). Tackling obesity in areas of high social deprivation: clinical effectiveness and cost-effectiveness of a task-based weight management group programme-a randomised controlled trial and economic evaluation. *Health Technology Assessment (Winchester, England)*, *20*(79), 1. | Patient-focused |
| 1. Kitchener, H. C., Gittins, M., Rivero-Arias, O., Tsiachristas, A., Cruickshank, M., Gray, A., ... & Roberts, C. (2016). A cluster randomised trial of strategies to increase cervical screening uptake at first invitation (STRATEGIC). *Health Technology Assessment (Winchester, England)*, *20*(68), 1. | Patient-focused |
| 1. Richards, D. A., Bower, P., Chew-Graham, C., Gask, L., Lovell, K., Cape, J., ... & Russell, A. (2016). Clinical effectiveness and cost-effectiveness of collaborative care for depression in UK primary care (CADET): a cluster randomised controlled trial. *Health Technology Assessment (Winchester, England)*, *20*(14), 1-192. | Other |
| 1. Littlewood, E., Duarte, A., Hewitt, C., Knowles, S., Palmer, S., Walker, S., ... & Gilbody, S. (2015). A randomised controlled trial of computerised cognitive behaviour therapy for the treatment of depression in primary care: the Randomised Evaluation of the Effectiveness and Acceptability of Computerised Therapy (REEACT) trial. *Health Technology Assessment (Winchester, England)*, *19*(101), 1. | Patient-focused |
| 1. Campbell, J. L., Fletcher, E., Britten, N., Green, C., Holt, T., Lattimer, V., ... & Warren, F. C. (2015). The clinical effectiveness and cost-effectiveness of telephone triage for managing same-day consultation requests in general practice: a cluster randomised controlled trial comparing general practitioner-led and nurse-led management systems with usual care (the ESTEEM trial). *Health technology assessment (Winchester, England)*, *19*(13), 1. | Other |
| 1. Ayling, K., Brown, M., Carlisle, S., Bennett, R., Buchanan, H., Dumbleton, J., ... & Vedhara, K. (2024). Optimizing mood prior to influenza vaccination in older adults: A three-arm randomized controlled trial. *Health Psychology*, *43*(2), 77. | Patient-focused |
| 1. Wright, C., Davey, A., Elmore, N., Carter, M., Mounce, L., Wilson, E., ... & Campbell, J. (2017). Patients’ use and views of real‐time feedback technology in general practice. *Health Expectations*, *20*(3), 419-433. | Other |
| 1. Burman, M., Zenner, D., Copas, A. J., Goscé, L., Haghparast-Bidgoli, H., White, P. J., ... & Kunst, H. (2024). Treatment of latent tuberculosis infection in migrants in primary care versus secondary care. *European Respiratory Journal*, *64*(5). | Other |
| 1. Santer, M., Muller, I., Becque, T., Stuart, B., Hooper, J., Steele, M., ... & Thomas, K. S. (2022). Eczema Care Online behavioural interventions to support self-care for children and young people: two independent, pragmatic, randomised controlled trials. *bmj*, *379*. | Patient-focused |
| 1. Farmer, A. J., Oke, J., Hardeman, W., Tucker, L., Sutton, S., Kinmonth, A. L., ... & Holman, R. R. (2016). The effect of a brief action planning intervention on adherence to double-blind study medication, compared to a standard trial protocol, in the Atorvastatin in Factorial with Omega EE90 Risk Reduction in Diabetes (AFORRD) clinical trial: A cluster randomised sub-study. *Diabetes research and clinical practice*, *120*, 56-64. | Patient-focused |
| 1. Khunti, K., Gillies, C. L., Dallosso, H., Brady, E. M., Gray, L. J., Kilgallen, G., ... & Davies, M. J. (2016). Assessment of response rates and yields for Two opportunistic Tools for Early detection of Non-diabetic hyperglycaemia and Diabetes (ATTEND). A randomised controlled trial and cost-effectiveness analysis. *Diabetes Research and Clinical Practice*, *118*, 12-20. | Patient-focused |
| 1. Yates, T., Edwardson, C. L., Henson, J., Gray, L. J., Ashra, N. B., Troughton, J., ... & Davies, M. J. (2017). Walking away from type 2 diabetes: a cluster randomized controlled trial. *Diabetic Medicine*, *34*(5), 698-707. | Patient-focused |
| 1. Billington, J., Coster, S., Murrells, T., & Norman, I. (2015). Evaluation of a nurse-led educational telephone intervention to support self-management of patients with chronic obstructive pulmonary disease: a randomized feasibility study. *COPD: Journal of Chronic Obstructive Pulmonary Disease*, *12*(4), 395-403. | Patient-focused |
| 1. Little, P., Stuart, B., Mullee, M., Thomas, T., Johnson, S., Leydon, G., ... & Moore, M. (2016). Effectiveness of steam inhalation and nasal irrigation for chronic or recurrent sinus symptoms in primary care: a pragmatic randomized controlled trial. *CMAJ*, *188*(13), 940-949. | Other |
| 1. Williamson, I., Vennik, J., Harnden, A., Voysey, M., Perera, R., Kelly, S., ... & Little, P. (2015). Effect of nasal balloon autoinflation in children with otitis media with effusion in primary care: an open randomized controlled trial. *Cmaj*, *187*(13), 961-969. | Other |
| 1. Rixon, L., Hirani, S. P., Cartwright, M., Beynon, M., Doll, H., Steventon, A., ... & Newman, S. P. (2017). A RCT of telehealth for COPD patient's quality of life: the whole system demonstrator evaluation. *The Clinical Respiratory Journal*, *11*(4), 459-469. | Other |
| 1. Retat, L., Pimpin, L., Webber, L., Jaccard, A., Lewis, A., Tearne, S., ... & Aveyard, P. (2019). Screening and brief intervention for obesity in primary care: cost-effectiveness analysis in the BWeL trial. *International Journal of Obesity*, *43*(10), 2066-2075. | Patient-focused |
| 1. Little, P., Read, R. C., Becque, T., Francis, N. A., Hay, A. D., Stuart, B., ... & Verheij, T. (2022). Antibiotics for lower respiratory tract infection in children presenting in primary care (ARTIC-PC): the predictive value of molecular testing. *Clinical Microbiology and Infection*, *28*(9), 1238-1244. | Other |
| 1. Tsivos, Z. L., Calam, R., Sanders, M. R., & Wittkowski, A. (2015). A pilot randomised controlled trial to evaluate the feasibility and acceptability of the Baby Triple P Positive Parenting Programme in mothers with postnatal depression. *Clinical child psychology and psychiatry*, *20*(4), 532-554 | Patient-focused |
| 1. Buszewicz, M., Griffin, M., McMahon, E. M., Walters, K., & King, M. (2016). Practice nurse-led proactive care for chronic depression in primary care: a randomised controlled trial. *The British Journal of Psychiatry*, *208*(4), 374-380. | Other |
| 1. Robb, K. A., Gatting, L., & Wardle, J. (2017). What impact do questionnaire length and monetary incentives have on mailed health psychology survey response?. *British journal of health psychology*, *22*(4), 671-685. | Patient-focused |
| 1. Landy, R., Hollingworth, T., Waller, J., Marlow, L. A., Rigney, J., Round, T., ... & Lim, A. W. (2022). Non-speculum sampling approaches for cervical screening in older women: randomised controlled trial. *British Journal of General Practice*, *72*(714), e26-e33. | Patient-focused |
| 1. Khunti, K., Highton, P. J., Waheed, G., Dallosso, H., Redman, E., Batt, M. E., ... & Yates, T. (2021). Promoting physical activity with self-management support for those with multimorbidity: a randomised controlled trial. *British Journal of General Practice*, *71*(713), e921-e930. | Patient-focused |
| 1. Laake, J. P., Vulkan, D., Quaife, S. L., Hamilton, W. T., Martins, T., Waller, J., ... & Duffy, S. W. (2021). Targeted encouragement of GP consultations for possible cancer symptoms: a randomised controlled trial. *British Journal of General Practice*, *71*(706), e339-e346. | Patient-focused |
| 1. Holt, T. A., Dalton, A. R., Kirkpatrick, S., Hislop, J., Marshall, T., Fay, M., ... & Hobbs, F. R. (2018). Barriers to a software reminder system for risk assessment of stroke in atrial fibrillation: a process evaluation of a cluster randomised trial in general practice. *British Journal of General Practice*, *68*(677), e844-e851. | GP-focused |
| 1. Neal, R. D., Barham, A., Bongard, E., Edwards, R. T., Fitzgibbon, J., Griffiths, G., ... & Hurt, C. N. (2017). Immediate chest X-ray for patients at risk of lung cancer presenting in primary care: randomised controlled feasibility trial. *British journal of cancer*, *116*(3), 293-302. | Other |
| 1. Harrison, P., Carr, E., Goldsmith, K., Young, A., Ashworth, M., Fennema, D., ... & Zahn, R. (2023). Antidepressant Advisor (ADeSS): a decision support system for antidepressant treatment for depression in UK primary care–a feasibility study. *BMJ open*, *13*(3), e060516. | GP-focused |
| 1. Wanat, M., Santillo, M., Galal, U., Davoudianfar, M., Bongard, E., Savic, S., ... & Tonkin-Crine, S. (2022). Mixed-methods evaluation of a behavioural intervention package to identify and amend incorrect penicillin allergy records in UK general practice. *BMJ open*, *12*(6), e057471. | GP-focused |
| 1. Ainsworth, B., Greenwell, K., Stuart, B., Raftery, J., Mair, F., Bruton, A., ... & Thomas, M. (2019). Feasibility trial of a digital self-management intervention ‘My Breathing Matters’ to improve asthma-related quality of life for UK primary care patients with asthma. *BMJ open*, *9*(11), e032465. | Patient-focused |
| 1. Anokye, N., Fox-Rushby, J., Sanghera, S., Cook, D. G., Limb, E., Furness, C., ... & Harris, T. (2018). Short-term and long-term cost-effectiveness of a pedometer-based exercise intervention in primary care: a within-trial analysis and beyond-trial modelling. *BMJ open*, *8*(10), e021978. | Patient-focused |
| 1. McManus, R. J., Little, P., Stuart, B., Morton, K., Raftery, J., Kelly, J., ... & Yardley, L. (2021). Home and Online Management and Evaluation of Blood Pressure (HOME BP) using a digital intervention in poorly controlled hypertension: randomised controlled trial. *bmj*, *372*. | Patient-focused |
| 1. Morrison, D., Wyke, S., Saunderson, K., McConnachie, A., Agur, K., Chaudhuri, R., ... & Mair, F. S. (2016). Findings from a pilot Randomised trial of an Asthma Internet Self-management Intervention (RAISIN). *BMJ open*, *6*(5), e009254. | Patient-focused |
| 1. Man, M. S., Chaplin, K., Mann, C., Bower, P., Brookes, S., Fitzpatrick, B., ... & Salisbury, C. (2016). Improving the management of multimorbidity in general practice: protocol of a cluster randomised controlled trial (The 3D Study). *BMJ open*, *6*(4), e011261. | Other |
| 1. Little, P., Stuart, B., Andreou, P., McDermott, L., Joseph, J., Mullee, M., ... & Yardley, L. (2016). Primary care randomised controlled trial of a tailored interactive website for the self-management of respiratory infections (Internet Doctor). *BMJ open*, *6*(4), e009769. | Patient-focused |
| 1. Kessler, D. S., MacNeill, S. J., Tallon, D., Lewis, G., Peters, T. J., Hollingworth, W., ... & Wiles, N. J. (2018). Mirtazapine added to SSRIs or SNRIs for treatment resistant depression in primary care: phase III randomised placebo controlled trial (MIR). *bmj*, *363*. | Other |
| 1. Astbury, N. M., Aveyard, P., Nickless, A., Hood, K., Corfield, K., Lowe, R., & Jebb, S. A. (2018). Doctor Referral of Overweight People to Low Energy total diet replacement Treatment (DROPLET): pragmatic randomised controlled trial. *bmj*, *362*. | Patient-focused |
| 1. Sidhu, M. S., Daley, A., Jordan, R., Coventry, P. A., Heneghan, C., Jowett, S., ... & Jolly, K. (2015). Patient self-management in primary care patients with mild COPD–protocol of a randomised controlled trial of telephone health coaching. *BMC pulmonary medicine*, *15*, 1-11. | Patient-focused |
| 1. Howell-Jones, R., Gold, N., Bowen, S., Bunten, A., Tan, K., Saei, A., ... & Chadborn, T. (2023). Can uptake of childhood influenza immunisation through schools and GP practices be increased through behaviourally-informed invitation letters and reminders: two pragmatic randomized controlled trials. *BMC Public Health*, *23*(1), 143. | Patient-focused |
| 1. Campbell, J., Vaghela, K., Rogers, S., Pyer, M., Simon, A., & Waller, J. (2018). Promoting prompt help-seeking for symptoms–assessing the impact of a gynaecological cancer leaflet on presentations to primary care: a record-based randomised control trial. *BMC Public Health*, *18*, 1-7 | Patient-focused |
| 1. White, K., Potter, R., Patel, S., Nichols, V. P., Haywood, K. L., Hee, S. W., ... & CHESS team. (2019). Chronic Headache Education and Self-management Study (CHESS)–a mixed method feasibility study to inform the design of a randomised controlled trial. *BMC Medical Research Methodology*, *19*, 1-11. | Patient-focused |
| 1. Brazil, K., Cardwell, C., Carter, G., Clarke, M., Corry, D. A. S., Fahey, T., ... & Doyle, F. (2022). Anticipatory care planning for community-dwelling older adults at risk of functional decline: a feasibility cluster randomized controlled trial. *BMC geriatrics*, *22*(1), 452. | Patient-focused |
| 1. Patel, M., James, K., Moss-Morris, R., Ashworth, M., Husain, M., Hotopf, M., ... & PRINCE Primary trial team Nicola Ferreira Katie Watts Richard Turner Alisia Carnemolla Jennifer Robertson Shinal Patel Philipp Frank Paige Fisher-Smith Abigale Childs Iris Mosweu Claire Willis Fabio Simiao. (2020). Integrated GP care for patients with persistent physical symptoms: feasibility cluster randomised trial. *BMC family practice*, *21*, 1-15. | Other |
| 1. Davies, M. J., Kristunas, C. A., Alshreef, A., Dixon, S., Eborall, H., Glab, A., ... & Gray, L. J. (2019). The impact of an intervention to increase uptake to structured self-management education for people with type 2 diabetes mellitus in primary care (the embedding package), compared to usual care, on glycaemic control: study protocol for a mixed methods study incorporating a wait-list cluster randomised controlled trial. *BMC Family Practice*, *20*, 1-15. | Patient-focused |
| 1. Willcox, M., Simpson, C., Wilding, S., Stuart, B., Soilemezi, D., Whitehead, A., ... & Moore, M. (2021). Pelargonium sidoides root extract for the treatment of acute cough due to lower respiratory tract infection in adults: a feasibility double-blind, placebo-controlled randomised trial. *BMC complementary medicine and therapies*, *21*, 1-16. | Other |
| 1. Daley, A. J., Thomas, A., Roalfe, A. K., Stokes‐Lampard, H., Coleman, S., Rees, M., ... & MacArthur, C. (2015). The effectiveness of exercise as treatment for vasomotor menopausal symptoms: randomised controlled trial. *BJOG: An International Journal of Obstetrics & Gynaecology*, *122*(4), 565-575. | Other |
| 1. Mallen, C. D., Nicholl, B. I., Lewis, M., Bartlam, B., Green, D., Jowett, S., ... & Peat, G. (2017). The effects of implementing a point-of-care electronic template to prompt routine anxiety and depression screening in patients consulting for osteoarthritis (the Primary Care Osteoarthritis Trial): a cluster randomised trial in primary care. *PLoS medicine*, 14(4), e1002273. | GP-focused |
| 1. Gawler, S., Skelton, D. A., Dinan-Young, S., Masud, T., Morris, R. W., Griffin, M., ... & Iliffe, S. (2016). Reducing falls among older people in general practice: The ProAct65+ exercise intervention trial. *Archives of gerontology and geriatrics*, *67*, 46-54. | Patient-focused |
| 1. Macfarlane, G. J., Beasley, M., Scott, N., Chong, H., McNamee, P., McBeth, J., ... & Lovell, K. (2021). Maintaining musculoskeletal health using a behavioural therapy approach: a population-based randomised controlled trial (the MAmMOTH Study). *Annals of the rheumatic diseases*, *80*(7), 903-911. | Patient-focused |
| 1. MacPherson, H., Tilbrook, H., Richmond, S., Woodman, J., Ballard, K., Atkin, K., ... & Watt, I. (2015). Alexander technique lessons or acupuncture sessions for persons with chronic neck pain: a randomized trial. *Annals of internal medicine*, *163*(9), 653-662. | Other |
| 1. Dangour, A. D., Allen, E., Clarke, R., Elbourne, D., Fletcher, A. E., Letley, L., ... & Mills, K. (2015). Effects of vitamin B-12 supplementation on neurologic and cognitive function in older people: a randomized controlled trial. *The American journal of clinical nutrition*, *102*(3), 639-647. | Other |
| 1. Coulton, S., Bland, M., Crosby, H., Dale, V., Drummond, C., Godfrey, C., ... & Wu, Q. (2017). Effectiveness and cost-effectiveness of opportunistic screening and stepped-care interventions for older alcohol users in primary care. *Alcohol and alcoholism*, *52*(6), 655-664. | Patient-focused |
| 1. Coulton, S., Dale, V., Deluca, P., Gilvarry, E., Godfrey, C., Kaner, E., ... & Heather, N. (2017). Screening for at-risk alcohol consumption in primary care: a randomized evaluation of screening approaches. *Alcohol and alcoholism*, *52*(3), 312-317. | Other |

***Patient-focused:** Interventions that are behavioral, educational, or informational, targeting patients. **GP-focused:** Interventions that are behavioral, educational, or informational, targeting general practitioners (GPs). **Other:** Studies evaluating new models of care, assessing the impact of new clinical guidelines, or contributing to research methodology.

**Note:** If multiple papers referred to the same trial (e.g., a study protocol and its corresponding results paper), only one was included. Papers published after 2015 were excluded if their primary findings had already been reported before 2015. **Primary care settings:** Studies were considered to be conducted in primary care if participant recruitment took place in GP practices and/or if the research involved GPs. Studies conducted in secondary care, specialized services (e.g., sexual health clinics, mental health services, physiotherapy, maternity care, nursing homes), pharmacies, or dental practices were excluded. However, exceptions were made for studies comparing models of care, such as those examining primary care versus secondary care. Studies conducted in multiple countries were excluded.
